# Supplementary material for: COMPOSITUM 1 contributes to the architectural simplification of barley inflorescence via meristem identity signals
Source: Nat Commun. 2020 Oct 12;11:5138. doi: 10.1038/s41467-020-18890-y (PMC7550572; doi:10.1038/s41467-020-18890-y)
Supplement: Supplementary file 1 — Supplementary Information [file 41467_2020_18890_MOESM1_ESM.pdf]

COMPOSITUM 1 contributes to the architectural simplification of barley inflorescence via meristem identity signals

Poursarebani *et al.*

Supplementary Note 1. TILLING analysis of *BdCOM1* in *Brachypodium*

Our TILLING analysis in *Brachypodium distachyon* revealed several mutations in the *BdCOM1* homolog of which two (M4. 5446; Q116* and M4. 8373; S146N) with predicted severe damages to the protein domain and function were phenotypically characterized (**Supplementary Data 3**). Hence, per M4 plants, only homozygous M5 plants either with mutant genotype aa (3 to 4 plants) or wild type bb (3 to 4 plants) were selected. Per M5 plants, 10 M6 plants were grown. The homozygote states of the corresponding M5 and M6 mutant family transmitted a defect in palea structure (**Fig. 6**). In which, we always observed a palea scissor-like structure in all florets in mutants, as it collapses easily due to external mechanical pressure. In contrast, palea in Wt never made such structure while applying same external hand-pressure. Histological analyses of the *Brachypodium* mutants' palea revealed no obvious change in cell expansion (**Fig. 6**).

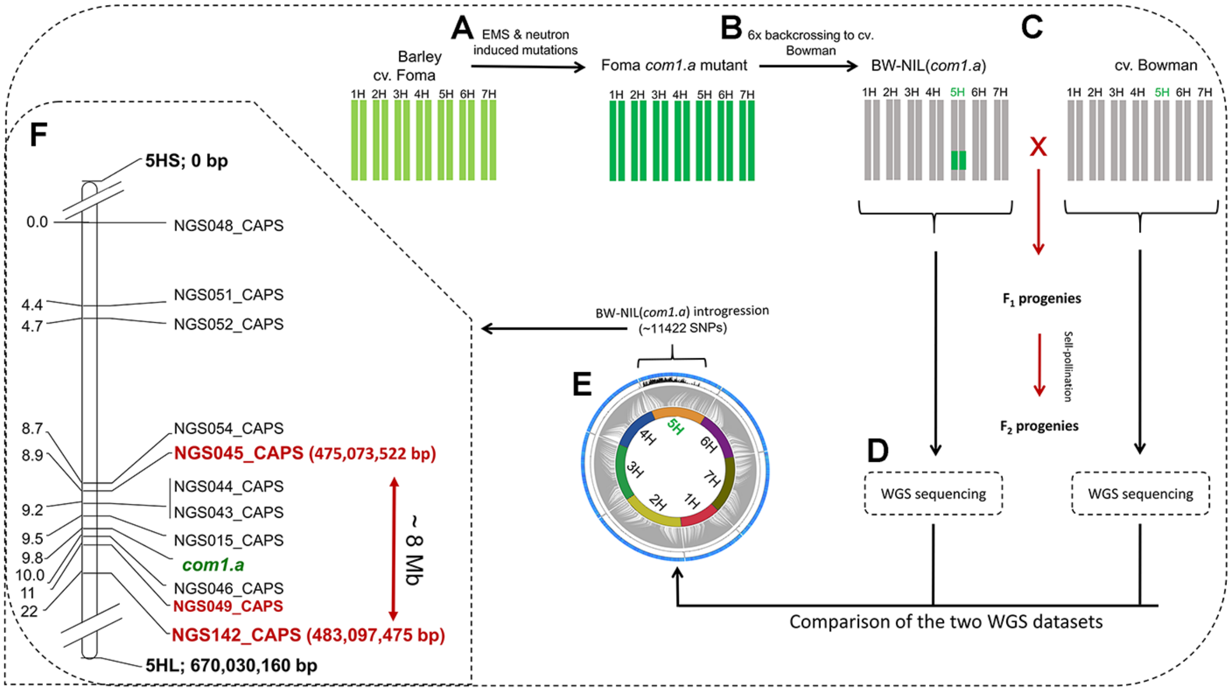

Supplementary Figure 1. Generation of induced Bowman near isogenic line (NIL), mapping population, marker resource and the low-resolution linkage map of *com1.a* in barley. (**A and B**) The *compositum1.a* phenotype was introduced (after its induction in barley cv. Foma) into a two-rowed barley cv. Bowman by six times backcrossing as reported previously [1]. Green area in chromosome 5H corresponds to the introgressed genomic segment carrying the underlying *com1.a* mutation. (**C**) The resulted BW near isogenic line (NIL) of *com1.a* allele; BW-NIL (*com1.a*), that is referred to as *com1.a* mutant in the current study, was crossed to cv. Bowman to generate F_2 population used in genetic mapping. (**D**) BW-NIL (*com1.a*) was whole genome shotgun (WGS) sequenced to 10x coverage (see Methods) and compared with already published WGS of Bowman [2]. (**E**) Represents alignment of the BW-NIL (*com1.a*) sequence assembly against the physically localized Bowman WGS sequence contigs. The corresponding SNPs (that were used for marker development) derived from the 5H introgression segment were plotted in the outer circle. (**F**) Genetic linkage mapping of *com1.a* in barley, derived from the F_2 mapping population (see part C). SNPs derived from E located within the introgressed region, were used for marker development. Markers in red were selected for high-resolution genetic mapping. A-B were performed and published previously [1]. C-F are entirely performed in the present study, except WGS of the wild type cv. Bowman.

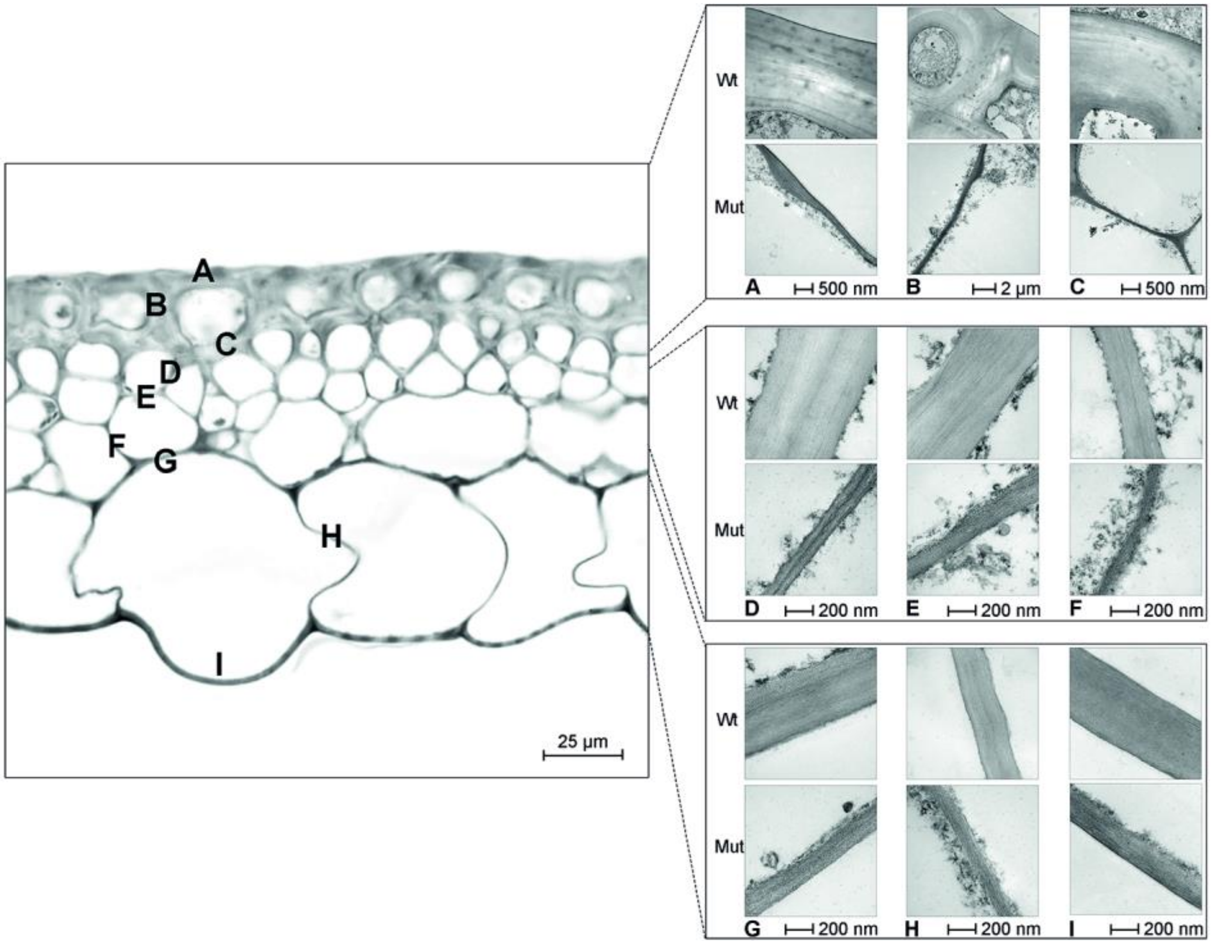

Supplementary Figure 2. TEM-based cell wall structure in wild type palea versus mutant *com1.a*. Left-side: cross section of wild type palea in which different cell positions (used to image the cell walls shown in right panel) across cell layers (see Fig. 1Y-Z for the layer IDs) are labeled. **Right-side**: cell wall thickness in Wt is depicted compared with that of mutant for each position of A-I; labeled accordingly in Left panel. BW-NIL (*com2.g*) mutant, that is visually similar in palea phenotype/structure with that of Wt cv. Bowman, were used here to compare with the mutant *com1.a*.

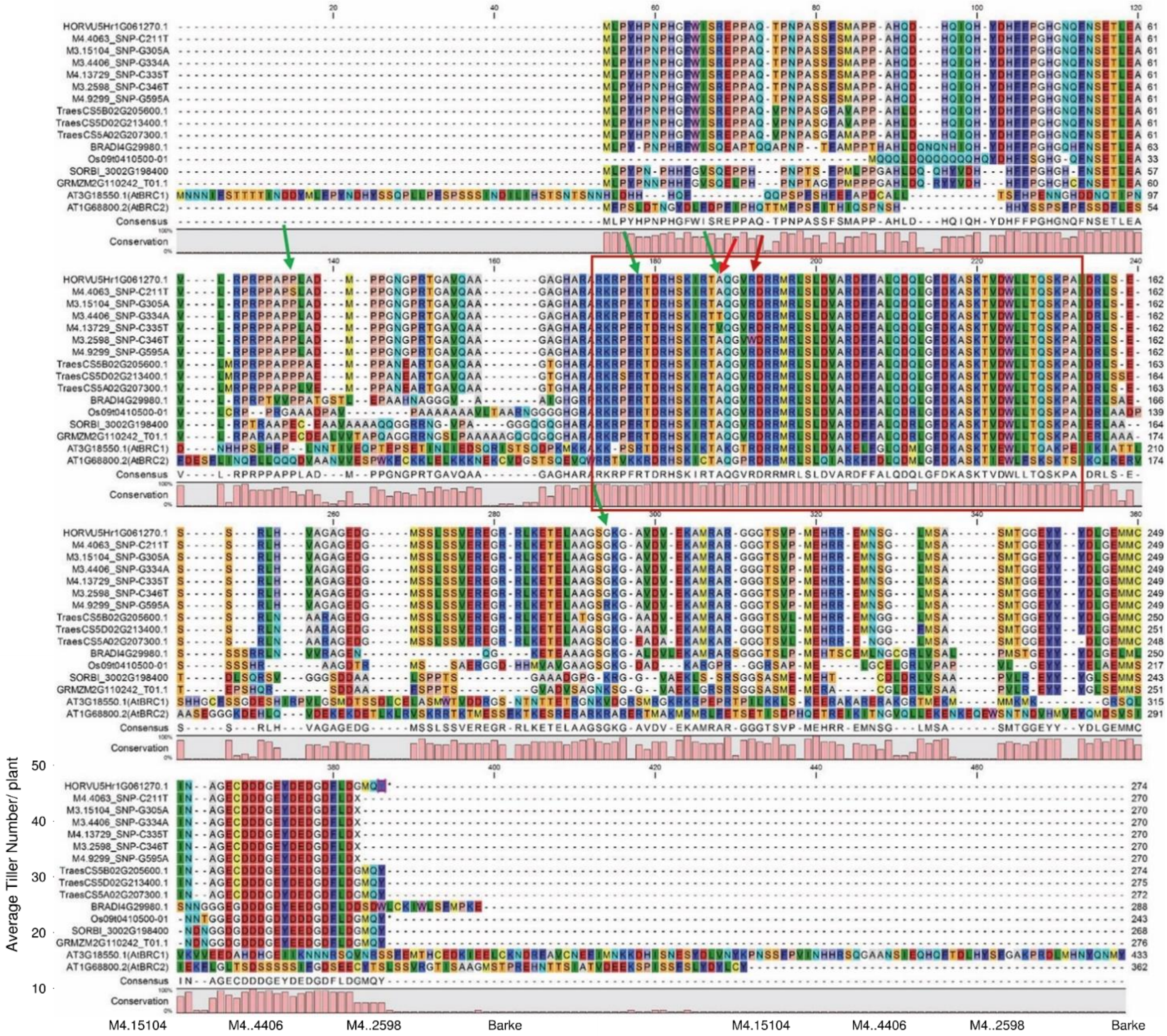

Supplementary Figure 3. Protein sequence alignment of the six barley TILLING lines. All six mutants displayed spike-branching (See Fig. 4). Four of the mutations were located within the corresponding TCP domain (the red frame). Mutations highlighted with red arrows show severe (dark red; M4.2598, conversion of a conserved aa R with electrically charged side chain to aa W with nonpolar side chain) and very mild (light red; M4.4406, conversion of A to T, a non-polar aa to polar one.) palea phenotypes. M4.13729 showed an aa change at the same position as M4.4406 but no palea phenotype was observed due to conversion of A to V; both from the same aa group of nonpolar side chain. M3.15104 also showed no palea phenotype as the conversion from R to K, is from the same group of basic aa with electrically charged side chain. The remaining two green arrows show mutations with no palea phenotype, perhaps because they are located outside of the TCP domain.

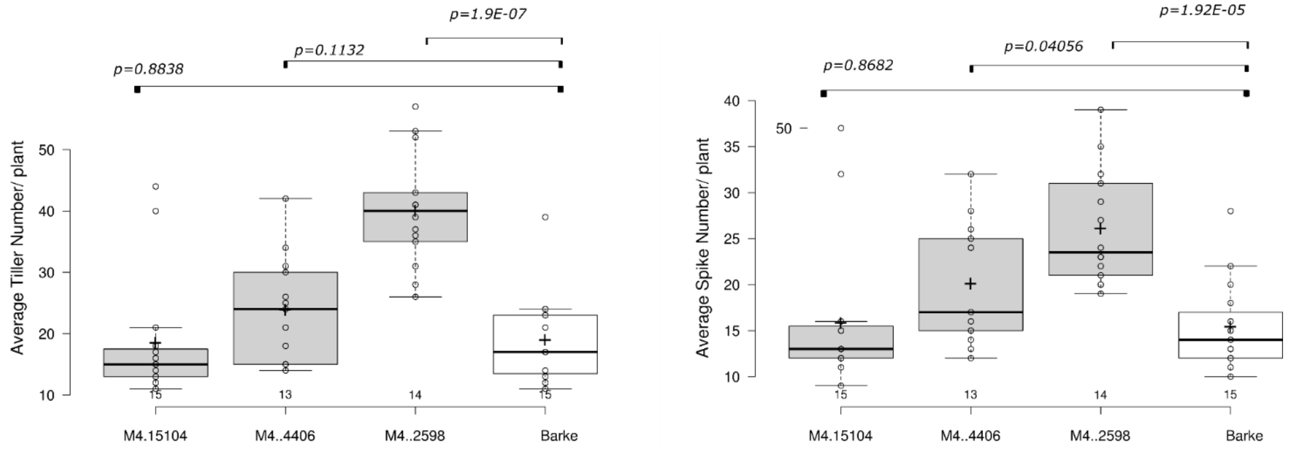

Supplementary Figure 4. Tillering related characters in barley TILLING lines. (A) Average tiller number and (**B**) spike number per plant of the three spike-branching barley TILLING lines are compared against wild type cv. Barke. This was performed to check whether *COM1* plays a role in tiller formation (**Fig. 7B**). Per box plot, plus signs show the mean while center bold lines show the medians; box limits indicate the 25th and 75th percentiles; whiskers extend 1.5 times the interquartile range from the 25th and 75th percentiles, outliers are represented by circles. A single experiment of twenty plants per genotype were performed under greenhouse conditions. *P* values were determined by using two-tailed paired Student's t-test. Source data are provided as a Source Data file.

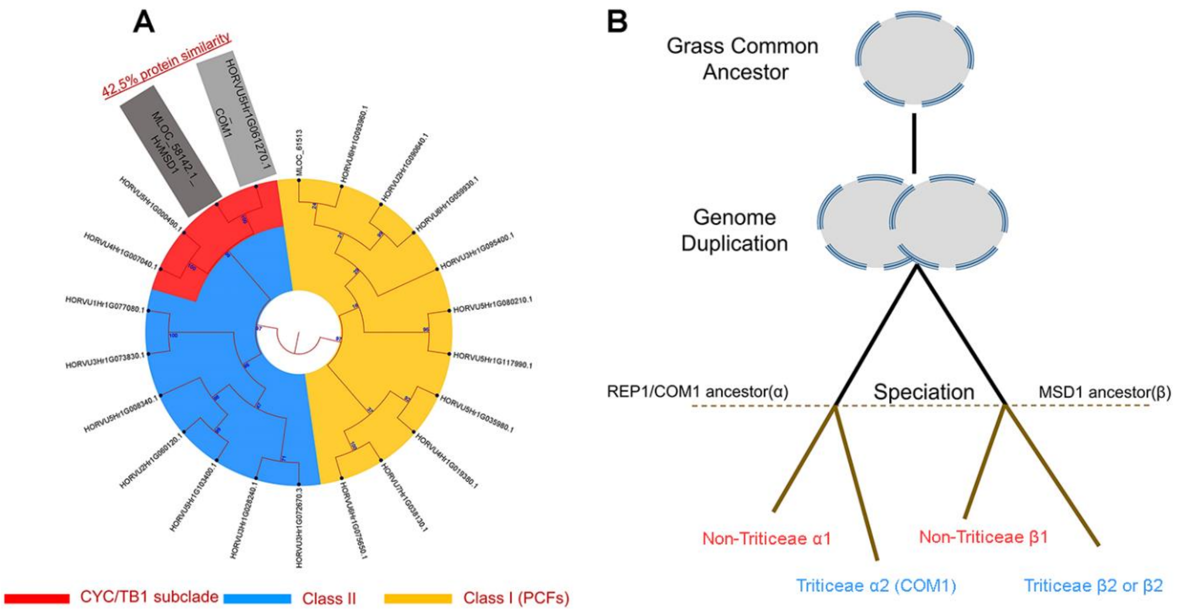

Supplementary Figure 5. Phylogeny of barley TCP proteins and the phylogeny-based model of *COM1* evolution. (A) 1,000 bootstraps based UPGMA phylogenetic tree of 22 barley TCP proteins. (**B).** Depicts origin and divergence of two (out-) paralogs of REP1/COM1 (α) and MSD1 (β*;* [3] clades from a common ancestor [4]. This divergence seems to happen after genome duplication in grass common ancestor and before the grass speciation (e.g. the separation of Triticeae from non-Triticeae species). More importantly, in case of paralogs α clade; although the clade members showed no after speciation duplication (no in-paralogy), except in maize (Fig. 2C; light-grey block), they have gone through a functional modification that separates the non-Triticeae function (α1) from that of the Triticeae subclade (α2). Coincidentally, the respective protein modifications separate inflorescence shapes between the two groups of grass plants by regulating contrasting phenotypes, i.e. formation versus inhibition of branches. Whether out-paralog β also differs in function among grasses (to be as β1 and β2) remains to be investigated. So far, only functional characterization of β in sorghum, the MSD1, has been reported [3]. Lines in brown represent the speciation process. Model depicted in **(B)** is reproduced from Studer and Robinson-Rechavi [5] with the kind permission from Elsevier.

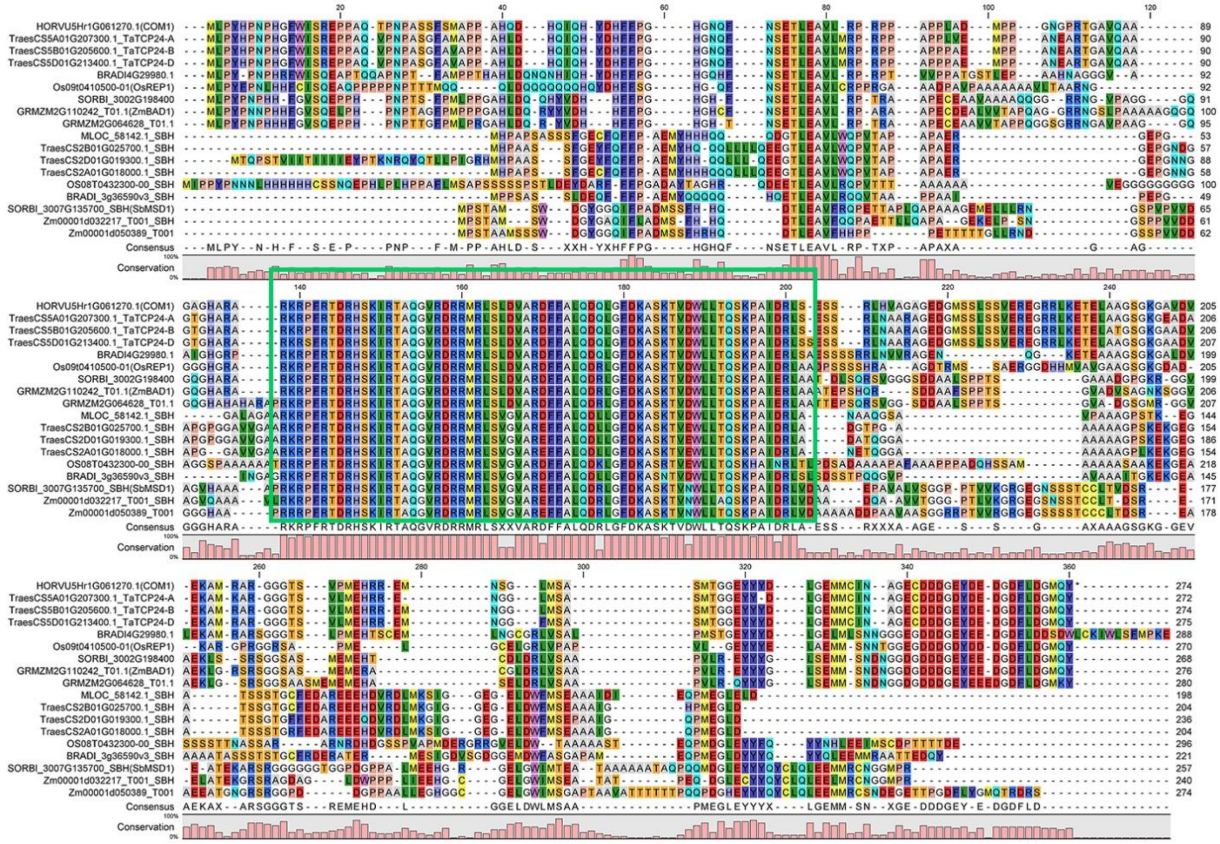

Supplementary Figure 6. Amino acid sequence alignment of the COM1 homologs among grasses. The green frame shows the conservation of the TCP domain.

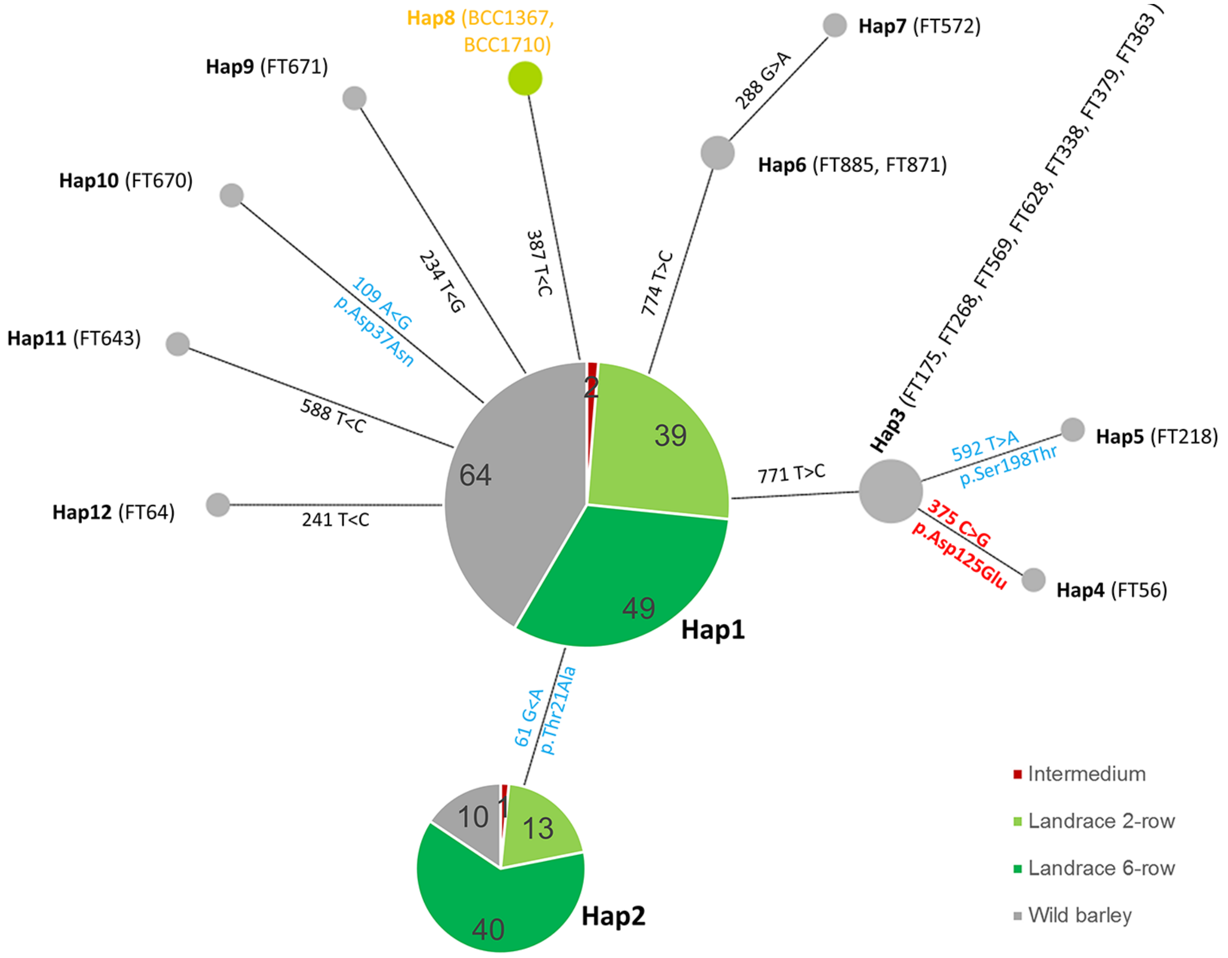

Supplementary Figure 7. Network analysis of *COM1*. Depicts grouping of 237 barley accessions (including 90 wild barleys) producing 12 haplotypes, comprising two main haplotypes: namely Hap1 (154 wild and landraces) and Hap2 (64 wild and landraces) with Hap1 being assigned as ancestral. The remaining 10 haplotypes, except Hap8 that contains two landraces, comprised only wild barleys (16 accessions) independently arisen from Hap1 during the course of evolution. Accessions representing each of the 12 haplotypes were grown and carefully inspected for the spike and palea phenotype but did not display any obvious phenotypic alteration. Sequences were obtained using primers for the CDS region. Source data are provided as a Source Data file.

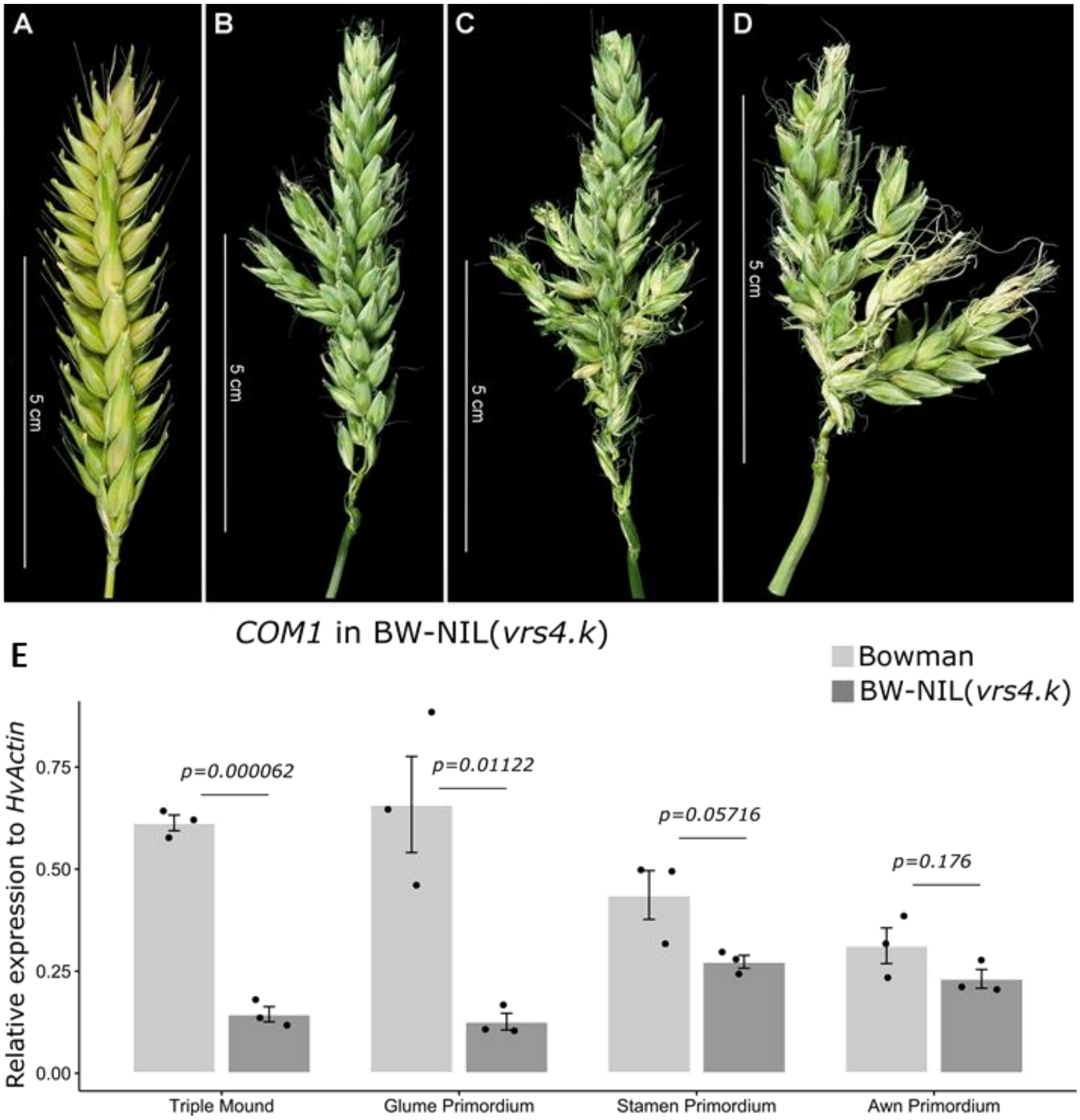

Supplementary Figure 8. Branch formation in *vrs4* mutant (*mul1.a*). (**A**) Mature spike of wild type progenitor cv. Montcalm with determinate triple spikelet meristem. (**B-D**) Mature spikes of *vrs4* mutant MC (*mul1.a*) showing various levels of branch proliferation at the spike base and middle portion of the spike. See also reference 6. **(E)** *COM1* transcripts in BW-NIL(*vrs4.k*) mutant. Mean ±SE of three biological replicates. *P* values were determined by using the two-tailed paired Student's t-test. This figure is reproduced from Poursarebani *et al*. [6] with the kind permission from Genetics Society of America.

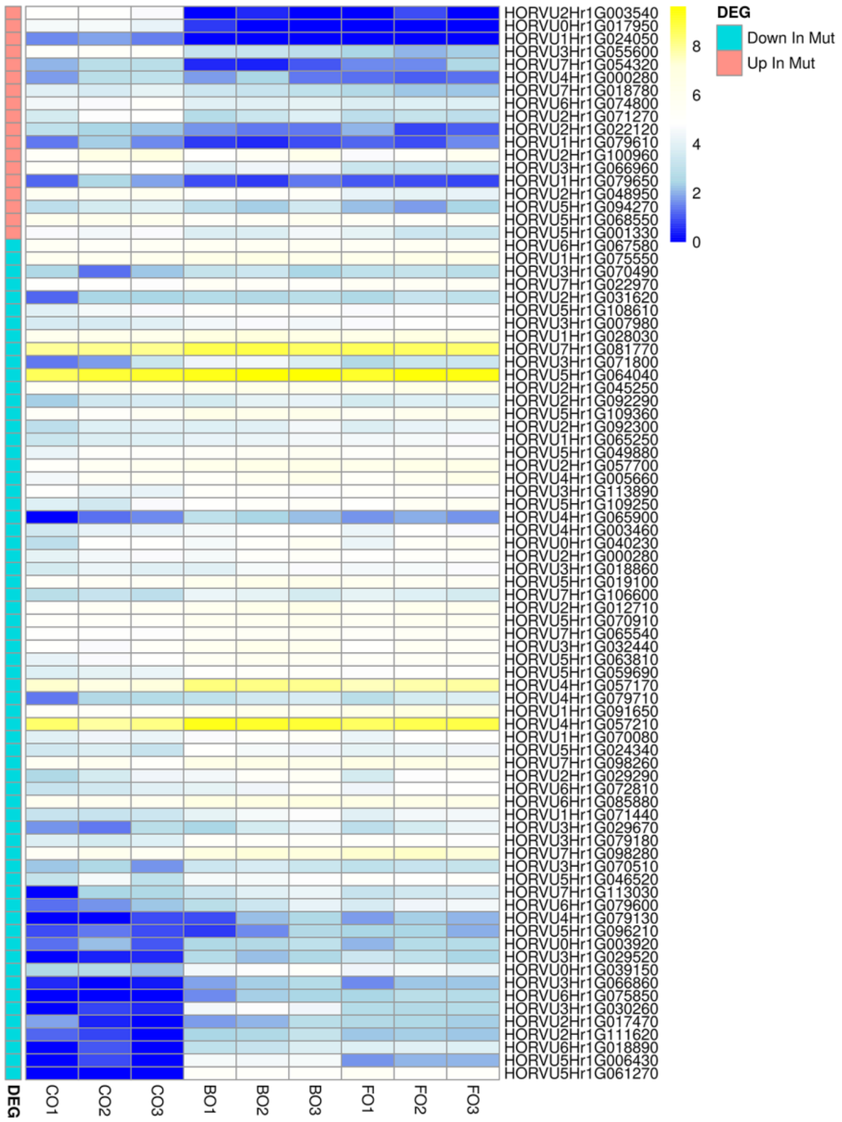

Supplementary Figure 9. Transcriptome analysis of *com1.a* using RNA seq. Heat map of all DE genes (found in RNA seq; n = 3 reps/3 stages /3 genotype) conjointly in the BW-NL(*com1.a*) as compared to the corresponding wild type cv. Bowman and cv. Foma. The scale bar at the top of the heat map indicates the transcript level of differentially regulated genes observed between wild type and mutant; blue color indicates down-regulation while red shows up-regulation. Transcript level was calculated using equation (1) (see Methods). One of the highly upregulated genes in mutant *com1.a* was HORVU3Hr1G055600 an NAD-dependent epimerase/dehydratase gene family associated with increased growth [7]. Other highly upregulated genes were involved in plant reproduction (HORVU7Hr1G054320, type I MADS-box transcription factor family protein [8,9]) as well as the floral meristem determinacy (HORVU7Hr1G018780; Homeobox-leucine zipper protein family protein 4; homology to HAT1 gene in *Arabidopsis thaliana*) [10]. Up regulation of these gene sets is fairly in line with the observed spike-branching phenotype of the mutant *com1.a*. See the main text for illustration of the relevant genes that are downregulated in mutant.

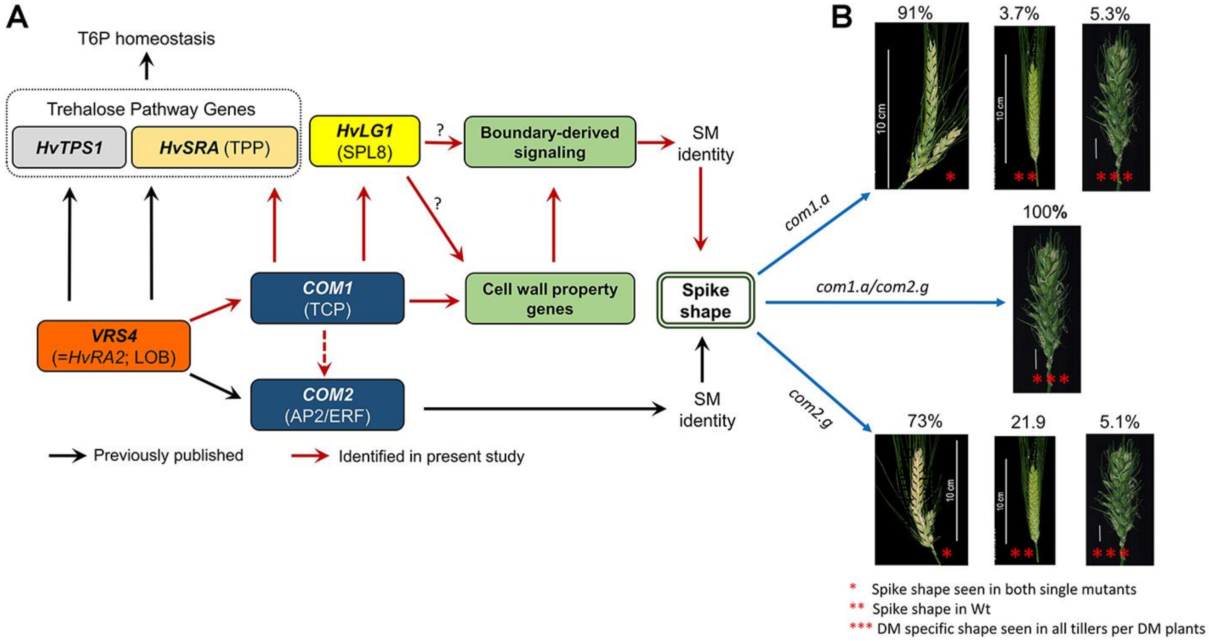

Supplementary Figure 10. The extended model of *COM1* transcriptional regulations. (**A**) Model of Wt *COM1* transcriptional regulations based on down-regulation of the Wt allele deduced either from RNAseq or the RT-qPCR results. Black arrows are interactions reported previously [6,11], while red arrows are detected in the current study. The red arrow connecting *VRS4* to *COM1* is deduced from RT-qPCR analysis in which *COM1* expression was low in BW-NI(*vrs4.k*) mutant immature spike as compared to Wt (see **Supplementary Fig. 8E**). The red dashed arrow connecting *COM1* to *COM2* is deduced from RT-qPCR analysis in which *COM2* expression was low in only one late stage in BW-NIL (*com1.a*) mutant immature spike as compared to Wt (**Fig. 8O**). The remaining red arrows are deduced from RNAseq performed in the current study (see **Fig. 9B**). (**B**) The resulted spike phenotype (% of spikes per mutant plant) due to the respective gene(s) loss-of-function. Data are based on a single greenhouse-condition experiment and on averages of 20 plants (390 to 540 spikes) per mutant class (*com1.a*, *com2.g* and the respective double mutants).

Supplementary Table 1. Graphical genotyping of the critical F2 recombinants used to develop F3 families.

Marker ID	4839	5176	5250	5403	5409	5443	5504	4953	5326	5483	5070	5045	174	5407	5488
NGS065	h	h	h	h	h	h	b	b	b	b	a	a	a	a	a
NGS066	h	h	h	h	h	h	b	b	b	b	a	a	a	a	a
NGS046	h	h	h	h	h	h	b	b	b	b	a	a	a	a	a
NGS083	h	h	h	h	h	h	b	b	b	b	a	a	a	a	a
d1652	h	h	h	h	h	h	b	b	b	b	a	a	a	a	a
NGS049	h	h	h	h	h	h	b	b	b	b	a	a	a	a	a
NGS084	h	h	h	h	h	h	b	b	b	b	a	a	a	a	a
NGS168	h	h	h	h	h	h	b	b	b	b	a	a	a	h	a
NGS169	h	b	b	h	h	h	b	b	h	b	h	h	h	h	a
NGS166	h	b	b	h	h	h	b	b	h	b	h	h	h	h	a
*com1.a/*Phenotype	c	c	c	c	c	c	c	c	c	c	c	c	c	c	a
NGS164	h	b	b	h	h	h	b	b	h	b	h	h	h	h	a
NGS163	h	b	b	h	h	h	b	b	h	b	h	h	h	h	a
NGS160	h	b	b	h	h	h	b	b	h	b	h	h	h	h	a
NGS158	b	b	b	h	h	h	b	h	h	b	h	h	h	h	a
NGS094	b	b	b	b	b	b	h	h	h	h	h	h	h	h	h
NGS111	b	b	b	b	b	b	h	h	h	h	h	h	h	h	h
NGS112	b	b	b	b	b	b	h	h	h	h	h	h	h	h	h
NGS099	b	b	b	b	b	b	h	h	h	h	h	h	h	h	h
NGS142	b	b	b	b	b	b	h	h	h	h	h	h	h	h	h

According to the Joinmap tool input requirements marker genotypes were classified as homozygous Wt AA (b), heterozygous Wt Aa (h) or homozygous mutant aa (a). At the plant phenotypic level, Wt and mutant plants were labeled as c or a, respectively.

Supplementary Table 2. Measurement of branch angle in the sorghum TILLING mutants using M6 generation.

TILLING Line ID_M4	DNA Position (from start codon)	Location within the gene	Number of plants _ all homozygous	No of nodes/panicle/ family	Average No of angle per nodes/panicle /family	Average angle size (degree) per nodes/panicle/ family	Grain formation
ARS180	A430G	within TCP domain	7	3 basal	10.1	5.2***	only 20-40 grains/panicle
ARS137	C577T	after TCP domain	5	3 basal	9.3	15.9	Complete fertility
Wt BT623	-	-	5	3 basal	8.1	10.95	Complete fertility

***: two-tailed paired t-test (*P*=1.31E-07)
Source data are provided as a Source Data file.

Supplementary Table 3. Measurement of branch number in the sorghum TILLING mutants using M5 generation.

TILLING line ID_M4	DNA position (from start codon)	Location within the gene	Number of plants _all homozygous	Average No of nodes [1] /panicle/ family	Average No of branch per nodes/panicle/family
ARS180	A430G	within TCP domain	8	12.0	4.3*
ARS137	C577T	after TCP domain	8	11.5	4.4
Wt BT623	-	-	7	10.0	5.1

[1] we refer to the rachis area where whorls of branches emerge.

*: two-tailed paired t-test (*P*=0.03886)

Source data are provided as a Source Data file.

Supplementary References

1. Druka, A. *et al.* Genetic dissection of barley morphology and development. *Plant Physiol* **155**, 617-27 (2011).
2. Mascher, M. *et al.* A chromosome conformation capture ordered sequence of the barley genome. *Nature* **544**, 427–433 (2017).
3. Jiao, Y.P. *et al.* MSD1 regulates pedicellate spikelet fertility in sorghum through the jasmonic acid pathway. *Nature Communications* **9**, 822 (2018).
4. Zhao, J. *et al.* Genome-wide identification and expression profiling of the TCP family genes in spike and grain development of wheat (*Triticum aestivum L.*). *Frontiers in Plant Science* **9**, 1282 (2018).
5 Studer, R.A. & Robinson-Rechavi, M. How confident can we be that orthologs are similar, but paralogs differ? *Trends in Genetics* **25**, 210-216 (2009).
6. Poursarebani, N. *et al.* The genetic basis of composite spike form in barley and 'Miracle-Wheat'. *Genetics* **201**, 155-165 (2015).
7. Cao, Y.Y. *et al.* Identification of differential expression genes in leaves of rice (*Oryza sativa* L.) in response to heat stress by cDNA-AFLP analysis. *Biomed Research International* **2013**, 576189 (2013).
8. Zhao, T. *et al.* Characterization and expression of 42 MADS-box genes in wheat (*Triticum aestivum L.*). *Molecular Genetics and Genomics* **276**, 334-350 (2006).
9. Masiero, S., Colombo, L., Grini, P.E., Schnittger, A. & Kater, M.M. The emerging importance of Type I MADS box transcription factors for plant reproduction. *Plant Cell* **23**, 865-872 (2011).
10. Kellogg, E.A. *et al.* Early inflorescence development in the grasses (*Poaceae*). *Frontiers in Plant Science* **4**, 250 (2013).

11. Koppolu, R. *et al. Six-rowed spike4* (*Vrs4*) controls spikelet determinacy and row-type in barley. *PNAS* **110**, 13198-13203 (2013).
