# Supplementary material for: COMPOSITUM 1 contributes to the architectural simplification of barley inflorescence via meristem identity signals
Source: Nat Commun. 2020 Oct 12;11:5138. doi: 10.1038/s41467-020-18890-y (PMC7550572; doi:10.1038/s41467-020-18890-y)
Supplement: Supplementary file 3 — Reporting Summary [file 41467_2020_18890_MOESM3_ESM.pdf]

nature research

Corresponding author(s): Naser Poursarebani & Thorsten Schnurbusch

Last updated by author(s): 17.02.2020

Reporting Summary

Nature Research wishes to improve the reproducibility of the work that we publish. This form provides structure for consistency and transparency in reporting. For further information on Nature Research policies, see Authors & Referees and the Editorial Policy Checklist .

Statistics

For all statistical analyses, confirm that the following items are present in the figure legend, table legend, main text, or Methods section.

n/a	Confirmed	
☐	☒	The exact sample size (*n*) for each experimental group/condition, given as a discrete number and unit of measurement
☐	☒	A statement on whether measurements were taken from distinct samples or whether the same sample was measured repeatedly
☐	☒	The statistical test(s) used AND whether they are one- or two-sided<br>*Only common tests should be described solely by name; describe more complex techniques in the Methods section.*
☐	☒	A description of all covariates tested
☐	☒	A description of any assumptions or corrections, such as tests of normality and adjustment for multiple comparisons
☐	☒	A full description of the statistical parameters including central tendency (e.g. means) or other basic estimates (e.g. regression coefficient) AND variation (e.g. standard deviation) or associated estimates of uncertainty (e.g. confidence intervals)
☐	☒	For null hypothesis testing, the test statistic (e.g. *F*, *t*, *r*) with confidence intervals, effect sizes, degrees of freedom and *P* value noted<br>*Give P values as exact values whenever suitable.*
☒	☐	For Bayesian analysis, information on the choice of priors and Markov chain Monte Carlo settings
☒	☐	For hierarchical and complex designs, identification of the appropriate level for tests and full reporting of outcomes
☒	☐	Estimates of effect sizes (e.g. Cohen's *d*, Pearson's *r*), indicating how they were calculated

Our web collection on statistics for biologists contains articles on many of the points above.

Software and code

Policy information about availability of computer code

Data collection	ImageJ Ver. 1.46r
Data analysis	ImageJ Ver. 1.46r; NEBcutter Ver.2.0; BatchPrimer3; SDS Ver.2.2; SALAD Ver.1; SIFT Ver. 5.2.2; CLC sequence viewer V7.8.1; TCS Ver; 1.21; Sequencher Ver. 5.2.2.3; Joinmap Ver; 4.0; salmon Ver.1.0.0 ; edgeR Ver. 3.22.5; featurecounts Ver.1.6.2; TopHAT Ver.2.1.1; BWA Ver. 0.5.9; Samtools Ver. 0.1.18; vcftools Ver. 0.1.17; Circos Ver. 0.52.

For manuscripts utilizing custom algorithms or software that are central to the research but not yet described in published literature, software must be made available to editors/reviewers. We strongly encourage code deposition in a community repository (e.g. GitHub). See the Nature Research guidelines for submitting code & software for further information.

Data

Policy information about availability of data

All manuscripts must include a data availability statement. This statement should provide the following information, where applicable:

- Accession codes, unique identifiers, or web links for publicly available datasets
- A list of figures that have associated raw data
- A description of any restrictions on data availability

provided within the main text under the data availability statement.

Field-specific reporting

Please select the one below that is the best fit for your research. If you are not sure, read the appropriate sections before making your selection.

☒ Life sciences ☐ Behavioural & social sciences ☐ Ecological, evolutionary & environmental sciences

Life sciences study design

All studies must disclose on these points even when the disclosure is negative.

Sample size	In case of transcript analysis such as RNAseq and qRT-PCR, each experiment was performed at least in three independent experiments; each containing 15-25 plants/samples per genotype per stage. This number of samples was required to obtain sufficient amount of meristematic tissues required for extraction of mRNA. We performed no other sample size calculation. The 15-25 plants were selected randomly from a larger group of plants grown in uniform growth conditions (see above). In case of representative experiments (such as micrographs), each replicate contains at least three samples from three independent plants.
Data exclusions	Relevant to our RNAseq: all genes that showed FPM of 0 across all 45 samples were excluded from expression level calculations.
Replication	All experiments were performed at least with three different replicates with two exceptions. Firstly, the phenotypic measurements presented in Fig. 6J-L, Fig. 8I-N as well as in supplementary Fig. 4. In these cases, we had only one experiment (replicate) as it is clearly mentioned within the respective legend. We believe this is enough to provide a rough estimation of how, for instance, double mutants look like in terms of grain characters as compared to each of the single mutants; because, our study is not about yield assessment. Or, in case of Fig. 6J-L one experiment was enough to support lack of pulvinus observed in SEM analysis of Brachypodium mutant.<br>One additional exception (in which only one experiment is performed as mentioned in the legend) is the data reported in Fig 7B; the COM1 expression across the length of the immature spike (IS), thus, only in IS-B, IS-C, and IS-A. We need to remind that the expression of COM1 within immature spike (IS) tissues is reported and confirmed multiple times in this manuscript. Nevertheless, in this particular experiment we wanted to provide a complementary view of COM1 expression pattern across the length of the IS. Thus, we believe one replication would be enough as this is not the main objective of our work and even without that all conclusions derived in the manuscript will stay strongly valid. If editor insists on minimum 3 biological replicates. The data can go out, too. Please let us know asap.
Randomization	All samples (individual plants or the corresponding organ/tissue) were randomly selected from a larger number of plants or organs/tissues. We have not used any tool for randomization.
Blinding	We performed Blinding while collecting phenotypic data points or performing the phenotypic measurements. For instance, we phenotyped individual plants within the population for spike-branching, measuring branch angle, or cell wall thickness analysis without considering the prior knowledge available about the genotype of that plant; whether that plant is Wt or mutant. We practiced this type of Blinding to eliminate any bias in our analysis.

Reporting for specific materials, systems and methods

We require information from authors about some types of materials, experimental systems and methods used in many studies. Here, indicate whether each material, system or method listed is relevant to your study. If you are not sure if a list item applies to your research, read the appropriate section before selecting a response.

Materials & experimental systems

n/a	Involved in the study
☒	☐ Antibodies
☒	☐ Eukaryotic cell lines
☒	☐ Palaeontology
☒	☐ Animals and other organisms
☒	☐ Human research participants
☒	☐ Clinical data

Methods

n/a	Involved in the study
☒	☐ ChIP-seq
☒	☐ Flow cytometry
☒	☐ MRI-based neuroimaging
