# Supplementary material for: COMPOSITUM 1 contributes to the architectural simplification of barley inflorescence via meristem identity signals
Source: Nat Commun. 2020 Oct 12;11:5138. doi: 10.1038/s41467-020-18890-y (PMC7550572; doi:10.1038/s41467-020-18890-y)
Supplement: Supplementary file 4 — Description of Additional Supplementary Files [file 41467_2020_18890_MOESM4_ESM.pdf]

Description of Additional Supplementary Files

File name: Supplementary Data 1

Description: List of the primers

File name: Supplementary Data 2

Description: Genotypic and phenotypic data of the F3 progenies

File name: Supplementary Data 3

Description: List of the TILLING as well as induced mutants per corresponding species
