# Supplementary material for: COMPOSITUM 1 contributes to the architectural simplification of barley inflorescence via meristem identity signals
Source: Nat Commun. 2020 Oct 12;11:5138. doi: 10.1038/s41467-020-18890-y (PMC7550572; doi:10.1038/s41467-020-18890-y)
Supplement: Supplementary file 2 — Peer Review File [file 41467_2020_18890_MOESM2_ESM.pdf]

Reviewers' comments:

Reviewer #1 (Remarks to the Author):

In this paper, Poursarebani et al clone and characterize a barley mutant, com1. They have clearly identified the gene disrupted in the com1 mutant, and have done a lot of work to characterize com1 mutants in barley, B. distachyon, and sorghum. I agree with most of the authors' conclusions, but have some comments for improvement.

Major comments:
• I'm not convinced that the authors can say that COM1 and COM2 are acting independently. The double mutant analysis presented in the supplemental data eliminates straight epistasis, but the data they present doesn't clearly establish additivity. I think they should soften their language in relation to COM1/COM2 independence.
• Line 210- line 218, starting with 'This expression pattern suggests involvement of barley COM1 in specification of the spikelet meristematic boundary.' The in situs presented in this paper, which support this statement, don't clearly establish boundary expression. The signal to noise ratio in these in situs is very high. In addition, I don't think that the authors have the data to say that this gene is involved in the 'formation of meristem identity signals'. This interpretation should be confined to the discussion, and the language in the discussion and abstract should be softened.

Minor comments:
• In Figure 1, there are no wild type SEMs shown. This makes interpretation of the mutant phenotype challenging.
• The authors use the term 'first best homolog' and 'second best homolog', which are confusing. I think that it is better to use existing terms. Specifically:
o The phylogenetic analysis clearly supports orthology between COM1, WAB1 and OsREP, the authors should just say that COM1 is the ortholog of WAB1 in maize, and REP1 in rice.
o Line 172: 'Instead, COM1 seems to be an out-paralog 21 of SBHs including the sorghum gene SbMSD1 (44.1% sequence similarity to COM1)'. I had to read this sentence a few times to understand what the authors were saying. I would suggest simplifying to 'COM1 is a paralog of SbMSD1'. In-paralogs and out-paralogs are clear from the phylogenetic analysis.
• Line 225: 'This suggests that barley COM1 underwent purifying natural selection most likely for maintaining inflorescence shape.' Although COM1 does appear to have been under selection, the authors cannot know that this selection was related to maintaining inflorescence shape.
• Throughout: Tribe, sub-family and family names should not be in italics (e.g. Oryzeae, Andropogoneae and Triticeae should all be un-italicized)
• Throughout: 'Dicot' should be 'eudicot'

Reviewer #2 (Remarks to the Author):

In this study, Poursarebani et al. describe a barley mutant in a gene encoding the TCP transcription factor, COMPOSITUM 1 (COM1), which confers a branched spike phenotype. Barley, as with other Triticeae species, normally forms a very simple, unbranched spike, with sessile, single-flowered spikelets formed directly off of the main axis. The genetics analyses performed to clone this locus were thorough and well-done/documented. While orthologs of COM1 have been well-characterized in non-Triticeae species maize and rice, the significance of this work lies within the apparent functional divergence of this grass-specific TF among Triticeae and non-Triticeae species. The authors presented strong phenotypic and genetic evidence to support this functional diversity. In addition, they show very little natural variation in this gene across barley diversity, suggesting it was a target of purifying selection.

Based on several lines of evidence, they authors hypothesized that in barley, COM1 regulates cell growth via regulation of cell wall properties in boundary cells, which confer signals for meristem identity. This is a different model for COM1 function in non-Triticeae species, where orthologs of COM1 function in boundary establishment.
There are a number of places in the manuscript that could use clarification, including a number of mistakes in figure labeling (or lack there of), etc. In addition, some of the bold claims made about functional roles of COM1 in barley are not clearly supported by data provided and the authors should address this. Below are some comments for enhancing the impact of this manuscript.

Overall, figures and legends could use revision for reader clarity as well as fixing numerous mistakes. Currently, figure legends are not adequately descriptive - in many places, neither are descriptions of figures in the main text. Some panels in the figures are difficult to read. For example, in Figure 3 the legend is so sparse that it is very difficult to follow. Why is there no corresponding mutant picture to go along with 3K? In the legend, the transition from 3K to 3L and M is also not clear. Brachy data are noted as (G-R) but there is no panel for R!
Also, there are mistakes in numbering in the Supplementary data - Supp figure 10 (Lines 233-234) is really Supp figure 11, etc.

In Figure 3B - Do the mutants in sorghum not make seed? It looks like the wild-type is mature and the mutant is immature. The weight of the seeds can impact branch angle. It is important to have matched stages for this picture, unless seed production is disrupted, which is not clear from the manuscript. Also, are there no quantitative data for the sorghum mutant phenotype? Are there more branches in the mutant?

Orthologs of COM1 in maize and rice have been well-characterized, however this is not apparent until well into the manuscript. It seems this should be at least referred to in the abstract or introduction. The authors also mention MSD1 in sorghum as a second best match, but this gene is quite divergent from COM1 in both sequence and function. I am not sure I understand the reasoning to mention this here.

COM2 is mentioned quite frequently in manuscript, including in the abstract, but the authors do not clearly discuss what COM2 encodes and whether it has functional divergence in other grasses too. I think this is a potential point of confusion for readers.
Related to this, I thought one of the most compelling results, as well as most impactful, was the analyses of the com1/com2 double mutants producing more grain number per spike. Is there aa reason why this was hidden in the Supplemental Data (Supp Figure 6) and not highlighted in the main manuscript?? Ideally there would be nice SEM comparisons of single mutants and wild-type alongside the double mutant developmental stages. The quantitative differences in yield traits is compelling!
In fact, many of the Supplemental Figures are much more high quality than some of the ones chosen for the main manuscript.

The in situs are a bit difficult to interpret due to what appears to be strong background and also suffer from poor explanations in the figure legend. While the described pattern in the boundary regions (Figure 4E-F) are sufficiently clear to me, I wonder if there is non-specific binding resulting from pooling of 3 different probes together. Is the sense control also a pool of 3 different sense probes? Perhaps the authors can comment on this.
Also, what is the expression of COM1 in the com1 mutant? Is it null or mis-expressed? Also, it is mentioned that COM1 acts in the same pathway as VRS4, LG1 and COM2 – it may help dissect this genetic pathway by performing in situs in these mutants?

Line 216 is a hypothesis for discussion. In my opinion, the statement seems too bold as written here and the evidence is not strong enough to support it.

The use of qRT-PCR in determining genetic pathways is unclear as described and not well documented in the Supplemental data.

Minor comments:
- Lines 91 and 95 – a better explanation of 'induced mutation' would be helpful
- Line 97 – not clear whether the allele in question was EMS or neutron-induced?
- Figure 1 F vs G confusing to readers in lines 96-100. The figure legend is clear, but in the text, it jumps between the mutant and the NIL and it is confusing to understand the morphological differences between them in the text.
- Line 103 – there is no text to specify that 1L shows the mutant phenotype
- (overall this descriptive section of Fig 1 in the text needs to be clearer for readers looking along at the figure – not all panels mentioned/specified)
- Also assuming Fig 1L and M are the mutant and not the NIL?
- 109-110 – what does this look like in a wild type? These images should be labeled in the figure.
- Better description of terminology for broader readership – line 115 – palea should be minimally described at least since it appears throughout the manuscript.
- Line 118-120: "Histological analyses using cross sections of paleae…were expanded in size and numbers". Did the authors actually measure the cell size and number? Was statistical testing done?
- Line 182 – there is no tassel in sorghum. Should read panicle or head.
- Line 187 – the additional vascular bundles also found in barley, no?
- Figure 2D – what do the numbers represent? Some more info as a legend in the figure or in the legend would be helpful. Overall it is difficult to read.
- Also Figure 5B is lacking enough description and is difficult to understand. What does the "transcript level" stand for? Did the authors arbitrarily set up the expression of one of the genes in the wildtype as 0 or 1?
- Page 12, line 250-251: "Among other significantly down-regulated genes in com1.a, we found important genes associated with cell wall properties and integrity (Fig. 5D).". there are no genes in Fig. 5D.

Reviewer #3 (Remarks to the Author):

The authors of this manuscript are studying barley inflorescence development. They have isolated the Compositum 1 (Com1) gene, showed that it encodes a TCP transcription factor and it that is expressed in inflorescence meristem boundaries. They also showed that the Com1 promotes branch formation in non triticeae grasses but branch inhibition in the triticeae. Finally, they proposed a pathway of vrs4 – com1 – lg1. This paper clearly provides novel information regarding inflorescence development and interesting models to test.

Comments:

1. The vrs4-com1-lg1 module that is proposed is consistent with the maize model but more data could be added to strengthen and elaborate on the model. I would like to see double mutant analysis and RNA in situ hybridization of each gene on the other mutants that are supportive of the model. For example, does the double mutant of Hvlg1 and com1 and RNA in situs of a lg1 probe on com1 mutant support the model?. More explanation is needed on page 11 lines 231-233 that describes that COM1 is downstream if VRS4.

2. More evidence needs to be presented that shows that com1 is grass specific. The phylogenetic tree that was presented is a small slice of the plant kingdom.

3. The RNA in situ hybridizations could be better. In particular, I do not see vascular bundles in Figure 4E described on page 10 line 207. Also, I am not sure why Fig 4F and G are included in the same

figure. They seem to show the complementary information and G has background and the signal is hard to see. I suggest a single image that shows the details.

4. In figure 3 there is no R as stated in the Figure legend.

5. I am not sure what is being referred to in last sentence of the abstract that says “This meristem identity module…” There is no description of the module in the abstract to refer to.

Reviewer #4 (Remarks to the Author):

This paper characterizes a TCP transcription factor, COMPOSITUM1, that is involved in specifying branch formation in Triticeae. The Triticeae are unusual among grasses in having an unbranched inflorescence so identifying a gene that appears to suppress branching is of general interest, both from the point of view of evolution but also of agronomy. The authors have a near-isogenic line that is a true null, apparently a deletion of COM1, and they have also located a set of other deletion mutants (shown in supplementary data) that have lost or have lesions in the same gene. Thus they provide good evidence that they have indeed cloned the COM1 locus.

The paper is interesting and analysis of this particular mutant could provide new insights into inflorescence development. I have two concerns about this paper however, the first having to do with the characterization of the com1 phenotype and the second having to do with organization and presentation.

1. The authors suggest that COM1 exerts its effects via control of cell structure. However, cell structure is only investigated in the palea. The anatomy and histology of the meristems, boundary region and branches are not described or shown. Line 99 states that the com1a NIL lacks a pulvinus. It would be helpful to have a photograph of the branch axil to show this. Sections through the axillary region are also important.

The paper is presented as though the reader is already familiar with the details of Hordeum inflorescence architecture. For example, Figure 1 nicely shows the development of the mutant but there is no comparison to a normal inflorescence. I suggest that the authors include images of normal barley development for comparison, and if possible reproduce images of com2.

Likewise, com2.g is an important component of this study but it needs a clearer introduction. It first appears in passing on lines 111-112, and then on line 153 where the authors discuss double mutants. If the reader is not familiar with the com2.g phenotype, this discussion is hard to interpret. One possible solution would be to add a paragraph to the introduction to describe the phenotype of the compositum mutants, indicate how many there are, and provide a short summary of their salient characteristics.

As with the description of com1.a mutant, the anatomy and histology of the double mutant needs much more careful description.

The palea phenotype is intriguing, but I question the interpretation that there is more sclerenchyma in the mutant. The cells in the layer labeled Scl are not particularly thick walled as would be expected of sclerenchyma. The mutant definitely has more layers of cells and there are certainly differences in the nature of the ad- and abaxial epidermis, but the description of the anatomy in lines 119-120 is not really accurate.

2. The paper is not well organized, which makes it difficult to follow. The short format requires that a lot of critical information be relegated to the supplemental data, and the authors have not worked out

how to make a coherent story out of the main text. The supplemental data is thus not truly supplemental but rather integral to the entire presentation and I had to keep flipping back and forth between the two just to be able to understand the data. For example, line 94 states "The Compositum-Barley com1.a (compositum 1.a) is an induced mutant with a branched spike introgressed into the two-rowed cv. Bowman (BW)…" This statement isn't fully accurate since it implies that the mutant somehow appeared in the introgression line. The next sentence plus Supplementary Fig. 1 are required to clarify that the Compositum-Barley com1.a was discovered in a mutagenized line of cv. Foma, backcrossing the mutant to Bowman led to the NIL, and that the term com1.a used throughout the text actually refers to the NIL.

Another organizational problem appears in line 181, where Sorghum is introduced without any preamble and apparently out of the blue. All other data presented to this point are from barley. The shift to sorghum requires some sort of transition and/or mention in the introduction to the paper.

Other specific comments:

Line 124 – This statement needs to be more cautious. COM1 could regulate cell growth via cell wall modifications but the two processes could also be independent phenotypes regulated by COM1. Also no evidence is presented of cellular differences in the branches of the com1a mutant vs. the wild type.

Lines 126-129 – Vascular bundle position and development are controlled in part by auxin, as are many aspects of meristem development. Thus the fact that COM1 affects vasculature in the palea as well as aspects of meristem identity could indicate that it operates via hormones.

Line 106 – I suggest omitting the term "floral reversion" since it implies that the meristem was specified as a flower but then reverses to an earlier identity.

Line 134 – This paragraph refers to com1.a, which originally was a mutation in cv. Foma and then introgressed into Bowman. In this paragraph are the authors discussing the original mutant or the Bowman NIL? Again the reader has to refer to the Supplementary information for the critical details. It would be clearer if line 134 simply described the cross as "Bowman wt x Bowman com1aNIL".

Fig. 2C – the phylogenetic tree would be easier to interpret if the species names were written out. The gene names can be placed elsewhere. I don't see a good reason to put the bootstrap values in blue; they can be black, which would also make them easier to read.

Lines 182-183 – The term tassel refers only to the staminate inflorescence in maize and is not generally applied to sorghum. The morphologial description of the sorghum inflorescence is also inaccurate. Sorghum has only one primary branch per node, but the internodes of the rachis do not all elongate equally so that branches appear to be whorled. Presumably these whorls of branches are what the authors call a "node"? What happens to the pulvinus in the sorghum plants?

Line 202 – The phrase "creating more complex inflorescence structures" is inaccurate. There is no change in lateral branch number in the mutants in rice or Brachypodium, and the number is apparently reduced in sorghum.

Line 211 – I do not understand the morphology being described here. The sentence implies that long branches in maize and sorghum fuse with each other and with the inflorescence meristem, which doesn't make a lot of sense. They appear to me to be quite separate.

Lines 213-216 – I am not convinced that the authors have data to support this model.

It is valuable to document that barley COM1 underwent purifying selection, but it is not unexpected. The level of purifying selection demonstrated is comparable to that of most transcription factors. This

point does not need much description or explanation.

Elizabeth A. Kellogg

RESPONSE TO THE REVIEWERS' COMMENTS;

Reviewer 1, Reviewer's Comments; In this paper, Poursarebani et al clone and characterize a barley mutant, com1. They have clearly identified the gene disrupted in the com1 mutant, and have done a lot of work to characterize com1 mutants in barley, B. distachyon, and sorghum. I agree with most of the authors' conclusions, but have some comments for improvement.

AUTHORS' RESPONSE; We deeply appreciated reviewer's comments that helped us to improve our manuscript. Please kindly see our responses below.

Major comments

Reviewer #1 Comment #1 (R1C1); I'm not convinced that the authors can say that COM1 and COM2 are acting independently. The double mutant analysis presented in the supplemental data eliminates straight epistasis, but the data they present doesn't clearly establish additivity. I think they should soften their language in relation to COM1/COM2 independence.

AUTHORS' RESPONSE; We thank the referee for this point. As it is correctly pointed out, our data eliminates epistasis as we have a clear over-performance of the double mutant as compared to each of the singles; both in generating meristematic tissues as well as in final grain production per spike. However, one could assume that COM2 might be partially and lately under the regulation of COM1. This is possible because there is a slight downregulation of COM2 in com1 mutant, but only in the later developmental stages of Stamen+Lemma primordia (Fig. 7O and the model in Fig. 8A; the dashed line red arrow from COM1 to COM2) that depict COM2 to be partly downstream of COM1. We consider this stage a late one, as compared to two stages earlier, the stage of triple mound, in which COM1 action is initiated. Thus, considering these facts, we agree that the actions of the genes are neither epistatic nor 100% independent. Thus, we believe the term 'partial independency' might provide a better interpretation of COM2 function as far as COM1 is concerned. Therefore, we replace the term 'independency' with the 'partial independency' for the com1 and com2 functional relation in the entire manuscript including the abstract. We have now moved the DM story completely to the main text as a separate section under the title; COM1 inhibits inflorescence branching partially independent to COM2.
Please also see our response to R2C12.

(R1C2); Line 210- line 218, starting with 'This expression pattern suggests involvement of barley COM1 in specification of the spikelet meristematic boundary.' The in situs presented in this paper, which support this statement, don't clearly establish boundary expression. The signal to noise ratio in these in situs is very high.

AUTHORS' RESPONSE; We thank the referee for raising this point. We sincerely would like to disagree that the expression pattern doesn't establish boundary expression. We still believe the boundary region to be the main area of COM1 expression. Please also see below supporting comment from Reviewer #2*; 'the described pattern in the boundary regions (Figure 4E-F) are sufficiently clear to me'*. However, we agree that the signal to noise ratio of in situ hybridization (ISH) might be high, and thus, we have re-performed this analysis and have provided now a clearer picture for COM1 ISH, please see Fig. 6. We would like to mention that detecting ISH signal for genes such as COM1 with very low level of expression is extremely challenging. During the course of our study, we have shown and personally discussed COM1 low level of expression, our ISH protocol and the difficulties of performing ISH for such genes with the experts in the field, including Prof. David Jackson, Cold Spring Harbor Laboratory. During his visit to our lab, he agreed with the challenge and suggested to use pools of COM1 probes to localize and visualize the expression. We found this idea very helpful as you can see also in the new experiment represented in Fig. 6.

(R1C3); In addition, I don't think that the authors have the data to say that this gene is involved in the 'formation of meristem identity signals'. This interpretation should be confined to the discussion, and the language in the discussion and abstract should be softened.

AUTHORS' RESPONSE; We appreciate the referee for providing a chance to clarify this point. The fact that COM1 is involved in the 'formation of meristem identity signals' is already supported by the com1 mutant branching-phenotype itself. It is clear from our SEM analysis of the mutant that the SM meristem is replaced with a different 'type' of IM-like meristem; thus, the destined SM has reversed to an earlier identity. In fact, the branching phenotype used to clone the gene is a visual proxy for this identity alteration/reversion. Thus, if com1 loss-of-function underlies branching it must be responsible—directly or indirectly— for the identity changes as well. So, we believe that lack of COM1 function has resulted in lack of forming proper boundaries/cell walls, resulting in lack of proper boundary/cell wall-derived signals and finally lack of proper SM identity. Since we believe that COM1 directly affects cell wall properties of boundary cells in the meristems, the lost identity providing signal is rather an indirect or

pleiotropic effect of the altered cell wall property. In any case, we think detecting those signals would be very exciting but is, however, beyond the scope of the current paper. We are planning to study this aspect in a follow-up work.

Minor comments;

(R1C4); In Figure 1, there are no wild type SEMs shown. This makes interpretation of the mutant phenotype challenging.

AUTHORS' RESPONSE; Thanks for this very useful comment. We added wild type images to this figure at the proper position. Please see new Fig. 1.

(R1C5); The authors use the term 'first best homolog' and 'second best homolog', which are confusing. I think that it is better to use existing terms. Specifically, o The phylogenetic analysis clearly supports orthology between COM1, WAB1 and OsREP, the authors should just say that COM1 is the ortholog of WAB1 in maize, and REP1 in rice.

AUTHORS' RESPONSE; We have corrected this statement as below;
"We searched for homologs and paralogs of COM1 in sequenced grass genomes, including rice, maize, sorghum, hexaploid wheat and *Brachypodium distachyon*, as well as *Arabidopsis thaliana* (**Fig. 2C**)".

(R1C6); Line 172; 'Instead, COM1 seems to be an out-paralog 21 of SBHs including the sorghum gene SbMSD1 (44.1% sequence similarity to COM1)'. I had to read this sentence a few times to understand what the authors were saying. I would suggest simplifying to 'COM1 is a paralog of SbMSD1'. In-paralogs and out-paralogs are clear from the phylogenetic analysis.

AUTHORS' RESPONSE; Considering the previous comment (R1C5) for which we corrected for FBH and SBH, we have revised the following statements to eliminate this confusion. Please see the corresponding results section, highlighted in yellow.
We are interested to keep the terms "in and out-paralogs" and also the corresponding citation in which these terms are very well illustrated. We believe this will help to enlighten that COM1 has no duplicated copy, which happened after-speciation, within the barley genome. And to make clear that we are comparing the COM1 with its true homolog from other genomes. We have seen a recently published paper that has done this mistake and has reported divergence by functionally comparing two closely

related genes; however, from different subclades! Furtheromore, to help clarifying the homology of COM1 to other grasses, we have peresneted the Supplementary Fig; now as Supplementary Fig. 5B.

(R1C7); Line 225; 'This suggests that barley COM1 underwent purifying natural selection most likely for maintaining inflorescence shape.' Although COM1 does appear to have been under selection, the authors cannot know that this selection was related to maintaining inflorescence shape.

AUTHORS' RESPONSE; Although, we cannot reject that there might be another character on which selection has been acted, this character must have been in tight linkage with the inflorescence shape. Therefore, selection has at least indirectly affected the shape of the inflorescence, thus we re-phrased our statements as below;
'This suggests that barley COM1 underwent purifying natural selection. We assume that this selection may have contributed to maintaining barley's slimmed-down inflorescence shape'
Please see also see the comment and our response to R4C18.

(R1C8); Throughout; Tribe, sub-family and family names should not be in italics (e.g. Oryzeae, Andropogoneae and Triticeae should all be un-italicized)

AUTHORS' RESPONSE; That's correct. Thanks for the clarification.

(R1C9); Throughout; 'Dicot' should be 'eudicot'

AUTHORS' RESPONSE; Sure, we changed to eudicot.

Reviewer #2 (Remarks to the Author);
In this study, Poursarebani et al. describe a barley mutant in a gene encoding the TCP transcription factor, COMPOSITUM 1 (COM1), which confers a branched spike phenotype. Barley, as with other Triticeae species, normally forms a very simple, unbranched spike, with sessile, single-flowered spikelets formed directly off of the main axis. The genetics analyses performed to clone this locus were thorough and well-done/documented. While orthologs of COM1 have been well-characterized in non-Triticeae species maize and rice, the significance of this work lies within the apparent functional divergence of this grass-specific TF among Triticeae and non-Triticeae species. The authors presented strong phenotypic and genetic evidence to support this functional diversity. In addition, they show very little natural variation in this gene across barley diversity, suggesting it was a target of purifying selection.

Based on several lines of evidence, they authors hypothesized that in barley, COM1 regulates cell growth via regulation of cell wall properties in boundary cells, which confer signals for meristem identity. This is a different model for COM1 function in non-Triticeae species, where orthologs of COM1 function in boundary establishment.

Reviewer #2 Comment #1 (R2C1); There are a number of places in the manuscript that could use clarification, including a number of mistakes in figure labeling (or lack there of), etc. In addition, some of the bold claims made about functional roles of COM1 in barley are not clearly supported by data provided and the authors should address this. Below are some comments for enhancing the impact of this manuscript.

AUTHORS' RESPONSE; We deeply appreciated reviewer's comments on the very detailed inspection of our manuscript. Please kindly see our responses below.

(R2C2); Overall, figures and legends could use revision for reader clarity as well as fixing numerous mistakes. Currently, figure legends are not adequately descriptive - in many places, neither are descriptions of figures in the main text.

AUTHORS' RESPONSE; Thanks for this comment. We have gone through all the figure legends and revised them again where needed. Please see highlighted areas in yellow within the legends for both main and supplementary figures.

(R2C3); Some panels in the figures are difficult to read. For example, in Figure 3 the legend is so sparse that it is very difficult to follow.

AUTHORS' RESPONSE; Thanks for this hint. Please see our response to the previous comment R2C2 and also please see highlighted area in yellow in Legend of Fig. 5 (previously named as Fig. 3)

(R2C4); Why is there no corresponding mutant picture to go along with 3K?

AUTHORS' RESPONSE;

We added a picture belonging to the mutant, please see Fig. 5L.

(R2C5); In the legend, the transition from 3K to 3L and M is also not clear.

AUTHORS' RESPONSE; We have changed the corresponding sentences in this legend. Please see Fig. 5, and the new legend.

(R2C6); Brachy data are noted as (G-R) but there is no panel for R!

AUTHORS' RESPONSE; This mistake is now corrected, and we have more images in this figure. Please see Fig. 5 and the legend.

(R2C7); Also, there are mistakes in numbering in the Supplementary data - Supp figure 10 (Lines 233-234) is really Supp figure 11, etc.

AUTHORS' RESPONSE; Thanks for the careful considerations. We have gone through all the figure legends and the Figs citation within the text and revised them again wherever needed. This paragraph is now revised as COM1-COM2 interaction is reported in a different section, and thus citations are clearer.

(R2C8); In Figure 3B - Do the mutants in sorghum not make seed? It looks like the wild-type is mature and the mutant is immature. The weight of the seeds can impact branch angle. It is important to have matched stages for this picture, unless seed production is disrupted, which is not clear from the manuscript. Also, are there no quantitative data for the sorghum mutant phenotype? Are there more branches in the mutant?

AUTHORS' RESPONSE; This is a valid point and we thank you for this comment. First of all, we have characterized grain production in the mutant and presented it partly in Table 1, and more intensively in Supplementary Table 5. The grain production of the mutant is disrupted apparently because of the problem in pollen formation as reported previously in rice (see; Zeng, D. et al. (2016). Genes & Genomics.) legend (Fig. 5A). Based on our visual inspection, the overall organization, dimension and angle size within the mutant, it is clear that it is independent of plant age. Therefore, we do not think that grain production could affect angle size in the sorghum mutant, e.g. by changing the angle due to grain weight. We have revised the corresponding legend (Fig. 5A) and added more illustration supported by data presented in Supplementary Table 5 to clarify this issue further. Furthermore, as expected there are fewer branches in mutant as presented in the main text as well as in the Supplementary Table 5.

(R2C9); Orthologs of COM1 in maize and rice have been well-characterized, however this is not apparent until well into the manuscript. It seems this should be at least referred to in the abstract or introduction.

AUTHORS' RESPONSE; Thanks for the comment. We added a short section to the last paragraph within the introduction to provide more advanced knowledge about the gene in other grasses including rice and maize. Please see the highlighted lines in yellow; the last paragraph of the introduction.

(R2C10); The authors also mention MSD1 in sorghum as a second-best match, but this gene is quite divergent from COM1 in both sequence and function. I am not sure I understand the reasoning to mention this here.

AUTHORS' RESPONSE; Thanks for this point. To this end, please see our response to (R1C5) and (R1C6)

(R2C11); COM2 is mentioned quite frequently in manuscript, including in the abstract, but the authors do not clearly discuss what COM2 encodes and whether it has functional divergence in other grasses too. I think this is a potential point of confusion for readers.

AUTHORS' RESPONSE; Thank you for making us aware about this important point. We have not talked about this gene as it was previously published and well described in our last publication. But we agree with this reviewer and thus have provided a short and sufficient info about this gene in the respective location in the last paragraph of the Introduction (highlighted in yellow) and in the double mutant section; a new section entitled by '*COM1* inhibits inflorescence branching partially independent to *COM2'*.

(R2C12); Related to this, I thought one of the most compelling results, as well as most impactful, was the analyses of the com1/com2 double mutants producing more grain number per spike. Is there aa reason why this was hidden in the Supplemental Data (Supp Figure 6) and not highlighted in the main manuscript?? Ideally there would be nice SEM comparisons of single mutants and wild type alongside the double mutant developmental stages. The quantitative differences in yield traits is compelling!

AUTHORS' RESPONSE; Thank you so much for this valid comment. We now moved all info regarding the com1/com2 double mutant from sup info and added it to the main text, as a separate section with an enhanced and clearer illustration of the DMs. In fact, we divided the section entitled by;

‘**COM1 encodes a TCP transcription factor that inhibits inflorescence branching independent of COM2’**, into two separate sections. One section is now for COM1 gene cloning entitled by ‘**COM1 encodes a class II, subclass CYC/TB1 TCP transcription factor’**. And a new section for DMs entitled by; ‘**COM1 inhibits inflorescence branching partially independent to COM2’**.

We have also made a new Fig that includes comparative SEM of singles with the double mutants at a comparable reproductive stage of immature spikes as well as clear Figs from spikes at maturity. see Fig. 7. Please also see our response to R1C1.

(R2C13); In fact, many of the Supplemental Figures are much more high quality than some of the ones chosen for the main manuscript.

AUTHORS’ RESPONSE; Although the referee has not specified/suggested which sup.fig to be moved to the main text, we have moved 3 different sup.figs to the main that are now as Fig3, 4 and 7. Please see our response also to R4C7 and R4C8.

(R2C14); The in situs are a bit difficult to interpret due to what appears to be strong background and also suffer from poor explanations in the figure legend.

AUTHORS’ RESPONSE; With regards to the background please see our reply to R1C2. We have revised and added the figure legend for the in situ work. Please see legend for the corresponding Fig, named now as Fig. 6

(R2C15); While the described pattern in the boundary regions (Figure 4E-F) are sufficiently clear to me, I wonder if there is non-specific binding resulting from pooling of 3 different probes together.

AUTHORS’ RESPONSE; Please see our reply to R1C2.

(R2C16); Is the sense control also a pool of 3 different sense probes? Perhaps the authors can comment on this.

AUTHORS’ RESPONSE; Yes, sense control was also pool of three sense control probes.

(R2C17); Also, what is the expression of COM1 in the com1 mutant? Is it null or mis-expressed?

AUTHORS' RESPONSE; We have not detected COM1 expression in the BW-NIL(com1.a) that is a deletion mutant using RNA seq. Except TILING mutants (for which COM1 expression was not checked), all other com1 mutants including BW-NIL(com1.a) are deletion mutants in which the entire gene is deleted.

(R2C18); Also, it is mentioned that COM1 acts in the same pathway as VRS4, LG1 and COM2 – it may help dissect this genetic pathway by performing in situs in these mutants?

AUTHORS' RESPONSE; mRNA in situs of *VRS4* and C*OM*2 are already available in our previous publication cited here in the current report. We have performed barley *LG1* in situ and added it to the paper as Fig. 8C-D. Barley *LG1* mRNA signals are largely overlapping with the expression domain of *COM1* signals of meristematic boundary regions.

(R2C19); Line 216 is a hypothesis for discussion. In my opinion, the statement seems too bold as written here and the evidence is not strong enough to support it.

AUTHORS' RESPONSE; We agree on this point. We removed the hypothetical statement from the result section. Section removed;
"In combination with our cell wall analysis in palea cells, this implies that barley COM1 may be involved in formation of meristem identity signals released from the boundary region through thickened cell walls encompassing boundary cells; thus, COM1 affects boundary signaling via cell wall modifications [23]. "

(R2C20); The use of qRT-PCR in determining genetic pathways is unclear as described and not well documented in the Supplemental data.

AUTHORS' RESPONSE; We have provided information to the legend of the Fig. 8 in which we have cited to the legend of supplementary Fig. 10; here we have provided comprehensive background info on each pathway (depicted as red arrows),

----

<u>Minor comments;</u>

(R2C21); - Lines 91 and 95 – a better explanation of 'induced mutation' would be helpful

AUTHORS' RESPONSE; We have revised the entire section under 'Atypical for Triticeae—barley *com1.a* mutant forms a branched inflorescence' and have added new info from sup note to here.

(R2C22); - Line 97 – not clear whether the allele in question was EMS or neutron-induced?

AUTHORS' RESPONSE; Both mutagenesis approaches have been used for creation of this mutant. We have clarified this point within the text.

(R2C23); - Figure 1 F vs G confusing to readers in lines 96-100. The figure legend is clear, but in the text, it jumps between the mutant and the NIL and it is confusing to understand the morphological differences between them in the text.

AUTHORS' RESPONSE; Sorry for this confusion. Both figures refer to BW-NIL(*com1.a*). Spikes represented as fig1.G belongs also to the mutant BW-NIL(*com1.a*). To avoid this confusion we keep only one fig, the fig.1F in which the more frequent and representing form of *com1.a* branching is displayed. We have made this point clear in the text that all analysis presented here are on the BW-NIL(*com1.a*) mutant and nothing is done on the original mutant, please see our reply to comment R2C21.

(R2C24); - Line 103 – there is no text to specify that 1L shows the mutant phenotype

AUTHORS' RESPONSE; We had not specifically addressed the 1L in the text; however, it has been addressed as Fig.1K-N (at the end of line 104) that includes 1L as well in the legend; Line 798. Nevertheless, we now have revised Fig. 1 and the legend. All images from this fig are now addressed accordingly within the text.

(R2C25); - (overall this descriptive section of Fig 1 in the text needs to be clearer for readers looking along at the figure – not all panels mentioned/specified)

AUTHORS' RESPONSE; As mentioned in the reply to comment R2C24, we now have revised Fig. 1 and the legend. All images from this fig are now addressed accordingly within the corresponding text/section.

(R2C26); - Also assuming Fig 1L and M are the mutant and not the NIL?

AUTHORS' RESPONSE; We have made this point clear in the text that all analysis presented here are on the BW-NIL(*com1.a*) mutant, please see first section of the results.

(R2C27); - 109-110 – what does this look like in a wild type? These images should be labeled in the figure.

AUTHORS' RESPONSE; Thanks for this useful comment. We added wild type images to this figure at the proper position. Please see Fig. 1.

(R2C28); - Better description of terminology for broader readership – line 115 – palea should be minimally described at least since it appears throughout the manuscript.

AUTHORS' RESPONSE; It is a very good point. We have added a short description for palea in beginning of this section entitled by 'COM1 restricts palea cell size by thickening their cell walls'.

(R2C29); - Line 118-120; "Histological analyses using cross sections of paleae…were expanded in size and numbers". Did the authors actually measure the cell size and number? Was statistical testing done?

AUTHORS' RESPONSE;

Since a clear difference in cell wall thickness between Wt and mutant cells became obsolete, we did not consider counting cell number. Cell size difference was clear by our visual inspection; Wt and mutant images are both taken at the same resolution and position clearly showing cell size differences (visually), most likely due to cell wall alterations. Thus, the claim is accordingly revised to avoid confusion, please see the corresponding results section, the lines highlighted in yellow.

(R2C30); - Line 182 – there is no tassel in sorghum. Should read panicle or head.

AUTHORS' RESPONSE; Thanks for this point. We have revised this accordingly.

(R2C31); - Line 187 – the additional vascular bundles also found in barley, no?

AUTHORS' RESPONSE; Yes, we thought referring to Table1 would be enough. However, we have added barley to this sentence now.

(R2C32); - Figure 2D – what do the numbers represent? Some more info as a legend in the figure or in the legend would be helpful. Overall it is difficult to read.

AUTHORS' RESPONSE; Thanks for this point. Each number refers to a motif type. Box with same number represent similar motifs. For instance, motif.1 (in light green) represents the TCP domain common across all genes. This illustration is now added to the legend.

(R2C33); - Also Figure 5B is lacking enough description and is difficult to understand. What does the "transcript level" stand for? Did the authors arbitrarily set up the expression of one of the genes in the wildtype as 0 or 1?

AUTHORS' RESPONSE; We have revised the description to provide sufficient information for this Figure. The calculation underlining the "transcript level" is now added to the legend. Thus, Transcript level= "Log(X+1)-Scaled Expression; where X is the normalized expression value of a given gene. +1 was considered to avoid problem with genes showing no expression!

The approach for analysis of the RNA seq data as well as for the detecting the DE genes are well described in the Math&Method section under the "Analysis of the RNAseq data". In short, Gene expression was estimated as read counts for each gene locus and fragment per million (FPM) values were extracted from the BWA-aligned reads. Expression levels were normalized by TMM method and p-values were then calculated. The Benjamini-Hochberg method was applied on the p-values to calculate q-values and to control the false discovery rate (FDR). Differentially expressed genes (DEGs) were defined as q-value < 0.05, log2 fold change > 1 or < -1.

(R2C34); - Page 12, line 250-251; "Among other significantly down-regulated genes in com1.a, we found important genes associated with cell wall properties and integrity (Fig. 5D).". there are no genes in Fig. 5D.

AUTHORS' RESPONSE; Thanks for this comment. We have corrected the figure citation that had to be Fig. 5B; now renamed as Fig. 8B.

Reviewer #3 (Remarks to the Author); The authors of this manuscript are studying barley inflorescence development. They have isolated the compositum 1 (COM1) gene, showed that it encodes a TCP transcription factor and it that is expressed in inflorescence meristem boundaries. They also showed that the COM1 promotes branch formation in non triticeae grasses but branch inhibition in the triticeae. Finally, they proposed a pathway of vrs4 – com1 – lg1. This paper clearly provides novel information regarding inflorescence development and interesting models to test.

AUTHORS' RESPONSE; We thank the referee for spending time assessing our work and for valuable comments. Please kindly see our responses below.

COMments;

Reviewer #3 Comment #1 (R3C1); 1. The vrs4-com1-lg1 module that is proposed is consistent with the maize model but more data could be added to strengthen and elaborate on the model. I would like to see double mutant analysis and RNA in situ hybridization of each gene on the other mutants that are supportive of the model. For example, does the double mutant of Hvlg1 and com1 and RNA in situs of a lg1 probe on com1 mutant support the model?

AUTHORS' RESPONSE; We thank the reviewer for these interesting points. First of all, we think that the data provided for this paper is sufficient to support our model of the gene interactions shown. We are planning to analyze the double mutants suggested here as a follow-up study. In case of in situ, we have performed barley *LG1* in situs on barley Wt plants (added now to the paper as Fig. 8C-B) nicely co-localizing with the *COM1* expression pattern. Secondly considering co-localization of *COM1* and *LG1* in barley, we already know that in situ of *LG1* in *com1* mutant would not provide additional info other than what have been showing in our RNA seq in which we have detected extreme downregulation of LG1 in com1 mutant. Moreover, *VRS4* (SM localized; Koppolu et al. 2013, PNAS) and *COM2* (glume primordium; Poursarebani et al. 2015, GENETICS) in situs have already been published. A barley *LG1* mutant is currently not available. Generating a new one may take a year of work and goes beyond the scope of this MS.

(R3C2); More explanation is needed on page 11 lines 231-233 that describes that COM1 is downstream if VRS4.

AUTHORS' RESPONSE; We have now provided more explanation and a clearer citation to the statement. Please see the corresponding section highlighted in yellow. Also, please also see our response to R2C7.

(R3C3); 2. More evidence needs to be presented that shows that com1 is grass specific. The phylogenetic tree that was presented is a small slice of the plant kingdom.

AUTHORS' RESPONSE; As we have also mentioned in the main text, the phylogenic analyzes of the TCP transcription have been reported multiple times (Bai et al. PNAS. 2012, Yuan et al. plant phys. 2008, Mondragon-Palomino et al. *Annals of Botany*. 2011, and Francis et al. Sci Rep. 2016 and some others) within which the COM1 orthologues are well depicted as being grass-specific; for instance in Fig S6 in Bai et al or Fig. 6 (group G1) in Francis etal. .
Thus, to not present redundant information we decided to only mention this fact in the main text along with referring to the related citations; mainly the maize paper describing the same gene being grass specific.

(R3C4); 3. The RNA in situ hybridizations could be better.

AUTHORS' RESPONSE; We thank the reviewer for this comment. We improved the images and added ISH work for *LG1*. Please also see our reply to comment **R1C2.**

(R3C5); In particular, I do not see vascular bundles in Figure 4E described on page 10 line 207.

AUTHORS' RESPONSE; Should be better now.

(R3C6); Also, I am not sure why Fig 4F and G are included in the same figure. They seem to show the complementary information and G has background and the signal is hard to see. I suggest a single image that shows the details.

AUTHORS' RESPONSE; We have included better and complementary images for in situ of COM1 in this figure. Please see now as Fig. 6.

(R3C7); 4. In figure 3 there is no R as stated in the Figure legend.

AUTHORS' RESPONSE; Thanks for this comment. We have corrected this mistake. Please see it as Fig. 5 now.

(R3C8); 5. I am not sure what is being referred to in last sentence of the abstract that says "This meristem identity module…" There is no description of the module in the abstract to refer to.

AUTHORS' RESPONSE; We changed the sentence to; "This meristem identity pathway has…"

Reviewer #4 (Remarks to the Author);

This paper characterizes a TCP transcription factor, COMPOSITUM1, that is involved in specifying branch formation in Triticeae. The Triticeae are unusual among grasses in having an unbranched inflorescence so identifying a gene that appears to suppress branching is of general interest, both from the point of view of evolution but also of agronomy. The authors have a near-isogenic line that is a true null, apparently a deletion of COM1, and they have also located a set of other deletion mutants (shown in supplementary data) that have lost or have lesions in the same gene. Thus, they provide good evidence that they have indeed cloned the COM1 locus. The paper is interesting and analysis of this particular mutant could provide new insights into inflorescence development. I have two concerns about this paper however, the first having to do with the characterization of the com1 phenotype and the second having to do with organization and presentation.

AUTHORS' RESPONSE; We deeply appreciate the referee for spending time evaluating our study and for providing detailed, very useful and valid comments, thank you. Please kindly see our responses below.

Reviewer #4 COMment #1 (R4C1); 1. The authors suggest that COM1 exerts its effects via control of cell structure. However, cell structure is only investigated in the palea. The anatomy and histology of the meristems, boundary region and branches are not described or shown.

AUTHORS' RESPONSE; Thanks for this critical comment that provides us with a chance to clarify this important point. As mentioned by the referee we have provided evidences through our anatomical visualization of palea cell wall properties that COM1 clearly effects cell structure, e.g. cell walls.

Assuming similar effects also in immature spike meristems at early reproductive stages; ‘visible and direct’ effects must be most likely limited to cells within the boundary region (IM-to-SM boundary) where COM1 is expressed. But, COM1’s ‘direct’ effect on other adjacent meristematic area, such as IM, BM, or SM, may not be easily visible/detectable, even when it is present.

Thus, the main objective would be to target the IM-to-SM boundary cells to analyze cell structural variations in the mutant versus Wt; like what we did for the palea. To this end, we need to be aware that boundary cells were shown to release local micro-mechanical signals into the adjacent meristems (e.g, for regulating meristem identity) by not only thickening their walls, but also by regulating cell wall elasticity (stiffness of the walls). Thus, we believe TEM analyses (that is to visualize wall thickness; as we did in palea) would not be informative, and thus, we would need other more specific tools to measure the cell wall elasticity as well. Because, we believe COM1 might exert its effects here by changes in cell wall stiffness rather than thickness. Such a detailed and local measurement of wall stiffness could be potentially measured by using AFM (Atomic Force Microscopy) or CFM (Cellular Force Microscopy) approaches. Unfortunately, however, due to technical limitations of AFM/CFM-based visualizations it may not be possible to detect any IM-to-SM boundary cells in any grass species. According to experts in the field (e.g. Dr. Arun Sampathkumar, Max-Planck Institute, Golm, Germany) with whom we have communicated, it is nearly impossible to find cell wall alterations due to the limited access of the AFM cantilever into highly curved domains of the boundary cells. This is one of the reasons one doesn't find much in the literature, or the scarcity of any published data on local cell wall stiffness (elastic modulus) along these regions. Moreover, with regards to the random occurrence of branching in the mutant (not all basal SMs will convert to IM-Like branches), a precise dissection-based sampling between mutant and Wt at synchronized growth condition and developmental stages is required. These requirements along with the limitations stated above, make this analysis far beyond the scope of this manuscript.

Alternatively, we have presented here our comparative RNAseq of immature spikes that provided a multi-layered understating of the gene regulatory system. Firstly, in our RNAseq we found a small number of extremely downregulated genes belonging, with no ambiguity, to cell wall structure. Secondly, observing such genes to be down-regulated in mutant spikes, and the indirect evidences from palea cells, was nicely in line with the currently available knowledge i.e. “wall of boundary cells can release micromechanical signals to affect property of the adjacent meristems (the respective review -papers are cited; Landrein et al. J Exp Bot, 2019 and Tamashige et al. Front. in Plant Science, 2015)”. More importantly, our RNAseq-based model of boundary cell wall alteration from inflorescence tissues explained very well the contrasting mutant phenotype in each of the species analyzed so far (Fig. 8E-H).

Thus, we think that our RNA seq data, in line with the structure of the palea cells (though only in analogy), provides enough support for our proposed model. However, we are still very much interested in

trying AFM, CFM, or some indirect measurements within the boundary regions but this is definitely beyond the scope of this paper. We therefore toned down our statements and adjusted the main title as well as the phrases used within the text accordingly.

(R4C2); Line 99 states that the com1a NIL lacks a pulvinus. It would be helpful to have a photograph of the branch axil to show this. Sections through the axillary region are also important.

AUTHORS' RESPONSE; We thank the reviewer for this valid point, we have now provided an image from the com1a NIL branch axil **(Fig. 1N**) and we also histologically analyzed sections through the axillary region for which the corresponding figure is also included (**Fig. 1M**).

(R4C3); The paper is presented as though the reader is already familiar with the details of Hordeum inflorescence architecture. For example, Figure 1 nicely shows the development of the mutant but there is no comparison to a normal inflorescence. I suggest that the authors include images of normal barley development for comparison, and if possible reproduce images of com2.

AUTHORS' RESPONSE; Thanks for this comment. We have added wilt type images to this Fig **(Fig. 1F-J)**. An image belong to the SEM analysis of the com2 mutant has also been added along with com1 and com1/com2 double mutants to provide the reader with an easy and immediate comparison **(Fig. 7)**.

(R4C4); Likewise, com2.g is an important component of this study but it needs a clearer introduction. It first appears in passing on lines 111-112, and then on line 153 where the authors discuss double mutants. If the reader is not familiar with the com2.g phenotype, this discussion is hard to interpret. One possible solution would be to add a paragraph to the introduction to describe the phenotype of the compositum mutants, indicate how many there are, and provide a short summary of their salient characteristics.

AUTHORS' RESPONSE; We thank the referee for this constructive comment. In the last part of the introduction, we have now added an introductory statement to all *compositum* mutants, shortly describing *com2* and then followed by *com1*, please see the last and new paragraph in the introduction. Please also see our response to R2C12.

(R4C5); As with the description of com1.a mutant, the anatomy and histology of the double mutant needs much more careful description.

AUTHORS' RESPONSE; We agree with this comment. We have now provided a very clear description of the DMs by providing a comparative illustration of DMs to each single mutant and to the Wt. This was based on both SEM based images of immature spikes as well as visual inspection of the spikes at maturity and the yield related characters. We have provided SEM comparisons of single mutants and wild-type alongside the double mutant at a comparable reproductive stage as well as at maturity. The DM section is now moved from Supp.info to the main text; please see our response to R2C12 as well as R1C1.

(R4C6); The palea phenotype is intriguing, but I question the interpretation that there is more sclerenchyma in the mutant. The cells in the layer labeled Scl are not particularly thick walled as would be expected of sclerenchyma. The mutant definitely has more layers of cells and there are certainly differences in the nature of the ad- and abaxial epidermis, but the description of the anatomy in lines 119-120 is not really accurate.

AUTHORS' RESPONSE; Wt and mutant images are both taken at the same resolution and position clearly showing cell size differences (visually), most likely due to cell wall differences. However, the claim questioned in this comment is accordingly revised to avoid confusion, please see the corresponding results section, the lines highlighted in yellow.

(R4C7); 2. The paper is not well organized, which makes it difficult to follow. The short format requires that a lot of critical information be relegated to the supplemental data, and the authors have not worked out how to make a coherent story out of the main text. The supplemental data is thus not truly supplemental but rather integral to the entire presentation and I had to keep flipping back and forth between the two just to be able to understand the data. For example, line 94 states "The compositum-Barley com1.a (compositum 1.a) is an induced mutant with a branched spike introgressed into the two-rowed cv. Bowman (BW)…" This statement isn't fully accurate since it implies that the mutant somehow appeared in the introgression line. The next sentence plus Supplementary Fig. 1 are required to clarify that the compositum-Barley com1.a was discovered in a mutagenized line of cv. Foma, backcrossing the mutant to Bowman led to the NIL, and that the term com1.a used throughout the text actually refers to the NIL.

AUTHORS' RESPONSE; We deeply thank the referee for this useful comment. We have reorganized the first result section in which we have included data from supplementary and revised the phrases for more convenient and clear-flow of the information provided. In particular, please see the first part of the

results in which we have elaborated the background info about the origin of the *com1* mutant for which Sup.Fig1 is cited for further clarification. All supplementary notes (except for Brachypodium) are moved to the main text. We have added the complete story of the DMs to the main text and some other minor but very critical revisions asked by other referees, please see the highlighted areas.

(R4C8); Another organizational problem appears in line 181, where Sorghum is introduced without any preamble and apparently out of the blue. All other data presented to this point are from barley. The shift to sorghum requires some sort of transition and/or mention in the introduction to the paper.

AUTHORS' RESPONSE; We agree with the referee and have provided a short and concise introductory statement within the Introduction of the paper (last paragraph, highlighted in yellow) as well as at the beginning of the part where we shifted to report the sorghum results.

Other specific comments;

(R4C8-1); Line 124 – This statement needs to be more cautious. COM1 could regulate cell growth via cell wall modifications but the two processes could also be independent phenotypes regulated by COM1.

AUTHORS' RESPONSE;

We understood that the referee is proposing that cell growth and cell wall modifications can be independent phenomena both regulated by COM1, thus the change in the cell growth could not necessary be the result in cell wall structural changes. Our statement was on the basis of the literature survey (line 120-121; Cell expansion is thought to be tightly linked to cell wall extensibility [13,14].) in which the two processes are shown to be largely correlated. However, we re-phrased the statement as below!

"Notably, mutant palea cells had clearly thinner cell wall structures, thus fewer mechanical obstructions for cell expansion, implicating that COM1 functions as a regulator of cell growth possibly via cell wall modifications."

(R4C8-2); Also no evidence is presented of cellular differences in the branches of the com1a mutant vs. the wild type.

AUTHORS' RESPONSE; We found this comment similar to the comment #1 of the referee #4; thus please see our previous response to R4C1.

(R4C9); Lines 126-129 – Vascular bundle position and development are controlled in part by auxin, as are many aspects of meristem development. Thus, the fact that COM1 affects vasculature in the palea as well as aspects of meristem identity could indicate that it operates via hormones.

AUTHORS' RESPONSE; We thank the referee for sharing this interesting idea. We agree with this point and think that *COM1* expression is regulated in part via hormones. Thus, we have already started an experiment to measure alteration in *COM1* expression by exogenously applying hormones such as auxin.

(R4C10); Line 106 – I suggest omitting the term "floral reversion" since it implies that the meristem was specified as a flower but then reverses to an earlier identity.

AUTHORS' RESPONSE; We thank the referee notifying this critical point. We have re-phrased the statement wherever mentioned and have used the term 'loss of identity' or an implication to that, instead.

(R4C11); Line 134 – This paragraph refers to com1.a, which originally was a mutation in cv. Foma and then introgressed into Bowman. In this paragraph are the authors discussing the original mutant or the Bowman NIL? Again the reader has to refer to the Supplementary information for the critical details. It would be clearer if line 134 simply described the cross as "Bowman wt x Bowman com1aNIL".

AUTHORS' RESPONSE; We rephrased the corresponding results section. Please see our response to the previous comment **R4C7**.

(R4C12); Fig. 2C – the phylogenetic tree would be easier to interpret if the species names were written out. The gene names can be placed elsewhere. I don't see a good reason to put the bootstrap values in blue; they can be black, which would also make them easier to read.

AUTHORS' RESPONSE; The Fig. 2C has now been revised accordingly. However, we kept the gene name as they included an abbreviation to the species IDs as prefix. Thus, a short description is added below the figure in which we clarified and cited each prefix to the corresponding species name. So, both gene ID and Species ID can be seen at the same figure.

(R4C13); Lines 182-183 – The term tassel refers only to the staminate inflorescence in maize and is not generally applied to sorghum. The morphologial description of the sorghum inflorescence is also inaccurate. Sorghum has only one primary branch per node, but the internodes of the rachis do not all

elongate equally so that branches appear to be whorled. Presumably these whorls of branches are what the authors call a “node”?

AUTHORS’ RESPONSE; We thank the reviewer for this informative and useful comment. We have provided a short description to the sorghum inflorescence in this paragraph. We replaced tassel with panicle. We revise the statement as below in which we define the term “node” with the term “whorls of branches” as reviewer explained.
“….and reduced primary branch number per “node” e.g. whorls of branches (5.4 in Wt vs. 4.2 in mutant, $P \leq 0.05$; **Supplementary Table 5**) ”

R4C14); What happens to the pulvinus in the sorghum plants?

AUTHORS’ RESPONSE; Thanks for asking this point. Our visual inspection showed that those pulvini are apparently absent or strongly reduced in size in the sorghum mutant (shown now in Fig. 5E-F). We would like to clarify that since it has been very well documented in the current study (in *Brachypodium*) and the past (in maize) that changes in branch angle is the result of changes in pulvinus size. To this end, we undertook branch angle measurements as a proxy for the size of the pulvinus. Thus in the case of sorghum, we did not analyze the pulvinus itself, e.g. measurement of pulvinus dimensions. Instead, as stated before, our visual inspection showed lack or reduced size of pulvini in the sorghum mutant.

(R4C15); Line 202 – The phrase “creating more complex inflorescence structures” is inaccurate. There is no change in lateral branch number in the mutants in rice or Brachypodium, and the number is apparently reduced in sorghum.

AUTHORS’ RESPONSE; We believe functional maize and sorghum *COM1* lead to having more branches and facilitate the generation of the pulvinus; thus, it at least contributes (if not doing everything alone) to the formation of two organs that are major components of a higher inflorescence complexity. The role of this gene in branch or pulvinus formation in rice is not known yet; however, we assume it is highly conserved and performs the same role as in maize and sorghum. In the case of Brachypodium, the gene has a kind of intermediate function (e.g. not changing the branch number, but pulvinus size), sitting between Triticeae and non-Triticeae. Such intermediary role resembles the Brachypodium phylogenic position that is considered to be an intermediate species between Triticeae and non-Triticeae. However, we agree with the reviewer and re-phrased our statement as bellow.

"In conclusion, COM1 homologs within non-Triticeae grasses primarily promote boundary formation and cell differentiation (as in rice palea)/proliferation (as seen for pulvinus) (Table 1); but similarly promote the formation of lateral axillary organs, e.g. branch or pulvinus, to contribute in maintaining complex inflorescence structures"

(R4C16); Line 211 – I do not understand the morphology being described here. The sentence implies that long branches in maize and sorghum fuse with each other and with the inflorescence meristem, which doesn't make a lot of sense. They appear to me to be quite separate.

AUTHORS' RESPONSE; Thanks for this comment. We understand the confusion! What we meant for maize was fusion of long branches (LB) to the inflorescence meristem (IM) that is in analogy with SM being fussed to IM in Triticeae. Of course, the LBs cannot be fused to each other in sorghum and maize. Thus, we re-phrased as below;

"However, since central and lateral spikelets do not fuse into each other or to the IM (as long branches do fuse to the IM in maize or sorghum), barley COM1 may not be involved in boundary formation *per se*."

(R4C17); Lines 213-216 – I am not convinced that the authors have data to support this model.

AUTHORS' RESPONSE; We thank the referee for this comment. We have removed this statement. Please also see our response to R2C19

(R4C18); It is valuable to document that barley COM1 underwent purifying selection, but it is not unexpected. The level of purifying selection demonstrated is comparable to that of most transcription factors. This point does not need much description or explanation.

AUTHORS' RESPONSE; Thanks for this comment. We agree with the referee. However, we are interested to keep this short illustration of an expected observation. However, we have modified the section title for the corresponding results as below to soften the language and to un-bold this finding. Thus, the new title; 'Barley COM1 function evolved to affect boundary signaling'

REVIEWERS' COMMENTS:

Reviewer #1 (Remarks to the Author):

This new version of the manuscript is much improved. All my comments have been dealt with, or I am satisfied with the authors' response. I am particularly impressed by the new in situ images - they look great!

Reviewer #2 (Remarks to the Author):

The authors have improved this manuscript significantly. The figures and legends are now much more intuitive and easy to follow through the text. Moving some of the supplemental figures up front really enhances the impact, and helps the reader follow the story better. Figure 7 is an impactful addition. The figure additions and revisions really improve the quality of the presentation of work.
The in situs were improved. I realize the complications with in situs in certain cases - but it is clear to me where the signal is. The last paragraph that was added to the introduction is also very helpful to put the work in perspective before going through the results.

They have also made appropriate edits to the text to clarify points, provide more detail, or soften some bold statements related to results. I believe the authors have addressed all of my comments and have made several other improvements based on comments from other reviewers. I really like the revised version of the manuscript.

One small typo I noticed reading through -
line 843 - 'imagining' should be imaging

Reviewer #3 (Remarks to the Author):

The authors have adequately addressed my and the other reviewers concerns.

Reviewer #4 (Remarks to the Author):

This version of the paper is much improved over the previous one. Including images of the wild type inflorescence makes the comparisons easier, and the colored SEMs in Figs. 1E-J are helpful for the reader who may not have all stages of barley development in the front of her mind. I also appreciate that the authors have introduced com2 to provide context to their comments in the manuscript; the new paragraph beginning on line 86 is very helpful for the reader. More details of the comparison to sorghum and Brachypodium are valuable additions. The many mutant alleles available provide convincing evidence that COM2 is indeed the proposed TCP transcription factor, and this evidence is much more clearly laid out in the current version of the paper.

Thanks for the explanation (in the rebuttal) of why cell walls could not be easily measured in the boundary domain. It makes sense that this could not be done for this paper.

The paper still has problems with writing and word choice. These are minor but numerous. I have not tried to correct the grammatical errors having to do with singular/plural, verb tense, and position of commas but these need to be addressed carefully. Other specific issues are as follows:

Line 262 - I am confused by the comments about the long branches “fusing” to the IM in maize and

rice. I've checked the original publications for BAD/WAB thinking I had forgotten some aspect of the phenotype but indeed the long branches are completely separate from the rachis of the inflorescence. I'm interpreting the word 'fused" to mean "inseparable" – i.e., with cellular continuity throughout such that the long branch appears as a ridge glued to the side of the inflorescence axis. Although such fusion does actually occur in short lateral branches of some Andropogoneae, I'm guessing that the authors must be using the word "fused" in a different way. Do they mean "completely upright", or "erect" or "non-spreading"?

Line 388 – "involvement of COM1 in printing such mechanical regulation" "printing" is the wrong word here. Maybe "promoting" or "directing"?

Lines 404-405 – The suggestion that the architecture of Triticeae inflorescences is a response to their temperate origin is an over-simplification. Triticeae is derived within the very large subfamily Pooideae, all of which are adapted to cool or cold climates (see multiple papers by Preston and Fjellheim) and many of which have highly branched inflorescences. The correlation of inflorescence morphology with climate is thus an artifact caused by comparing only a couple of cool climate species (wheat, barley) with a handful of warm climate species (sorghum, rice, maize). The authors cite Kellogg et al. 2013, which addresses phyllotaxis of the primary branches, rather than subsequent formation of higher order branches; I think the authors may have misinterpreted it. That publication notes that in subfamily Pooideae (of which Triticeae is a part), the primary branches of the inflorescence form in two ranks, rather than a spiral as in most other subfamilies. However, in most Pooideae those primary branches go on to form several higher orders of branches – i.e., the mature inflorescence is not a spike as in Triticeae but rather a branched panicle (e.g. oats) superficially similar to that in rice or sorghum. For this particular paper, most of this nuance is not relevant; the simplest route would be to avoid trying to correlate the unbranched inflorescence with climate.

Line 454. The text mentions Fig. 3G but I think this may be referring to what is now Fig. 5H.

Elizabeth A. Kellogg

AUTHORS RESPONSES TO THE REVIEWERS' COMMENTS.

Reviewer #1 (Remarks to the Author):

This new version of the manuscript is much improved. All my comments have been dealt with, or I am satisfied with the authors' response. I am particularly impressed by the new in situ images - they look great!

AUTHORS’ RESPONSE;

We thank the referee for the great contribution in enhancing the impact of our work.

Reviewer #2 (Remarks to the Author):

The authors have improved this manuscript significantly. The figures and legends are now much more intuitive and easy to follow through the text. Moving some of the supplemental figures up front really enhances the impact, and helps the reader follow the story better. Figure 7 is an impactful addition. The figure additions and revisions really improve the quality of the presentation of work.

The in situs were improved. I realize the complications with in situs in certain cases - but it is clear to me where the signal is. The last paragraph that was added to the introduction is also very helpful to put the work in perspective before going through the results.

They have also made appropriate edits to the text to clarify points, provide more detail, or soften some bold statements related to results. I believe the authors have addressed all of my comments and have made several other improvements based on comments from other reviewers. I really like the revised version of the manuscript.

One small typo I noticed reading through -

Reviewer2 Comment1 (R2C1): line 843 - 'imagining' should be imaging

AUTHORS’ RESPONSE;

We thank the referee for the extremely helpful comments in the first revision that improved the impact of our work.

The point made in the current comment is revised now accordingly, thank you.

Reviewer #3 (Remarks to the Author):

The authors have adequately addressed my and the other reviewers concerns.

AUTHORS’ RESPONSE;

We thank the referee for the valuable comments and the contribution in enhancing the impact of our work.

Reviewer #4 (Remarks to the Author):

This version of the paper is much improved over the previous one. Including images of the wild type inflorescence makes the comparisons easier, and the colored SEMs in Figs. 1E-J are helpful for the reader who may not have all stages of barley development in the front of her mind. I also appreciate that the authors have introduced com2 to provide context to their comments in the manuscript; the new paragraph beginning on line 86 is very helpful for the reader. More details of the comparison to sorghum and Brachypodium are valuable additions. The many mutant alleles available provide convincing evidence that COM2 is indeed the proposed TCP transcription factor, and this evidence is much more clearly laid out in the current version of the paper.

Thanks for the explanation (in the rebuttal) of why cell walls could not be easily measured in the boundary domain. It makes sense that this could not be done for this paper.

R4C1: The paper still has problems with writing and word choice. These are minor but numerous. I have not tried to correct the grammatical errors having to do with singular/plural, verb tense, and position of commas but these need to be addressed carefully.

AUTHORS' RESPONSE;

We thank the referee for her great contribution and the valuable comments that enhanced the impact of our work significantly. Considering the current comment, we would like to mention that we have sent our article to the language editing service prior to the first submission. However, we have gone through the text again to revise possible errors mentioned by the referee, please see Track Changes.

Other specific issues are as follows:

R4C2: Line 262 - I am confused by the comments about the long branches "fusing" to the IM in maize and rice. I've checked the original publications for BAD/WAB thinking I had forgotten some aspect of the phenotype but indeed the long branches are completely separate from the rachis of the inflorescence. I'm interpreting the word 'fused" to mean "inseparable" – i.e., with cellular continuity throughout such that the long branch appears as a ridge glued to the side of the inflorescence axis. Although such fusion does actually occur in short lateral branches of some Andropogoneae, I'm guessing that the authors must be using the word "fused" in a different way. Do they mean "completely upright", or "erect" or "non-spreading"?

AUTHORS' RESPONSE;

We would like to clarify this in the following statement; we are referring here to the maize and sorghum mutant plants in which the number of branches is reduced; and thus, we are here not referring to the Wt plants. In this case, it was clearly reported for the maize mutant that it has a lower number of branches! Now, how to explain this lower number of branches knowing the fact that the respective gene is putatively involved in the IM-to-BM boundary formation? We concluded for maize that the branches are

reduced in number perhaps because the formation of initial BM cells is lacking. ‘Boundary formation’ i.e. differentiation of the IM into initial BM cells- has not happened (most likely due to the absence of boundary initiation and signaling).Thus, cells that were supposed to become a lateral BM could not emerge. As a result, cells remain peripheral, not distinguishable anymore from the surrounding cells. Whether the lack of such cell differentiation can be considered as a “fusion” or “consumption” or “dissipation of branches” is hard to tell. We decided to use the term “fused” simply because the cells destined to become BM cells are still there. However, as a consequence of the lacking boundary initiation these cells still remain peripheral to the IM but without branch formation. We were scratching our heads about the wording, too. So, if you have a better term for the described events, we are happy to include it. We hope we could now clarify this.

Nevertheless, to reduce confusion we added the word ‘mutant’ to the statement to clarify that we are referring to the scenario in which the gene is mutated and not to the Wt allele. Here is the revised statement:

“However, since central and lateral spikelets do not fuse into each other or to the IM in the barley *com1* mutant (as long branches do fuse to the IM in maize or sorghum mutants; i.e. lowering the branch number per mutant IM), barley COM1 may not be involved in boundary formation per se but perhaps rather in boundary signaling (see below transcriptional results and discussion).”

R4C3: Line 388 – “involvement of COM1 in printing such mechanical regulation” “printing” is the wrong word here. Maybe “promoting” or “directing”?

AUTHORS’ RESPONSE;

Thanks for this suggestion. We replaced with the word “directing”.

R4C4: Lines 404-405 – The suggestion that the architecture of Triticeae inflorescences is a response to their temperate origin is an over-simplification. Triticeae is derived within the very large subfamily Pooideae, all of which are adapted to cool or cold climates (see multiple papers by Preston and Fjellheim) and many of which have highly branched inflorescences. The correlation of inflorescence morphology with climate is thus an artifact caused by comparing only a couple of cool climate species (wheat, barley) with a handful of warm climate species (sorghum, rice, maize). The authors cite Kellogg et al. 2013, which addresses phyllotaxis of the primary branches, rather than subsequent formation of higher order branches; I think the authors may have misinterpreted it. That publication notes that in subfamily Pooideae (of which Triticeae is a part), the primary branches of the inflorescence form in two ranks, rather than a spiral as in most other subfamilies. However, in most Pooideae those primary branches go on to form several higher orders of branches – i.e., the mature inflorescence is not a spike as in Triticeae but rather a branched panicle (e.g. oats) superficially similar to that in rice or sorghum. For this particular paper, most of this nuance is not relevant; the simplest route would be to avoid trying to correlate the unbranched inflorescence with climate.

AUTHORS’ RESPONSE;

We deeply appreciate the reviewer for this enlightening comment. We have revised the corresponding statement to avoid the misinterpretation pointed-out by the referee; however, we would like to keep the connection of TCP-TF responses to external conditions. Here is the new statement:

“Thus, the barley floral reductionism (from compound spike to spike shape; Fig. 1A-D) contributed by COM1, might be a response to yet unknown ecological factor(s) attributed to the origin of Triticeae.”

R4C5: Line 454. The text mentions Fig. 3G but I think this may be referring to what is now Fig. 5H.

AUTHORS’ RESPONSE;

This is revised now accordingly.

Naser Poursarebani & Thorsten Schnurbusch on behalf of all other co-Authors.
